# Blue Phosphorescence and Hyperluminescence Generated from Imidazo[4,5‐b]pyridin‐2‐ylidene‐Based Iridium(III) Phosphors

**DOI:** 10.1002/advs.202201150

**Published:** 2022-07-13

**Authors:** Xilin Yang, Xiuwen Zhou, Ye‐Xin Zhang, Deli Li, Chensen Li, Caifa You, Tai‐Che Chou, Shi‐Jian Su, Pi‐Tai Chou, Yun Chi

**Affiliations:** ^1^ State Key Laboratory of Luminescent Materials and Devices and Institute of Polymer Optoelectronic Materials and Devices South China University of Technology Guangzhou 510640 China; ^2^ School of Mathematics and Physics The University of Queensland Brisbane Queensland 4072 Australia; ^3^ Suzhou Joysun Advanced Materials Co., Ltd. Suzhou Jiangsu 215126 China; ^4^ Department of Chemistry Department of Materials Sciences and Engineering and Center of Super‐Diamond and Advanced Films (COSDAF) City University of Hong Kong Hong Kong SAR 999077 China; ^5^ Department of Chemistry National Taiwan University Taipei 10617 Taiwan

**Keywords:** carbene, cyclometalate, hyperphosphorescence, iridium, organic light‐emitting diode

## Abstract

Four isomeric, homoleptic iridium(III) metal complexes bearing 5‐(trifluoromethyl)imidazo[4,5‐b]pyridin‐2‐ylidene and 6‐(trifluoromethyl)imidazo[4,5‐b]pyridin‐2‐ylidene‐based cyclometalating chelates are successfully synthesized. The meridional isomers can be converted to facial isomers through acid induced isomerization. The *m*‐isomers display a relatively broadened and red‐shifted emission, while *f*‐isomers exhibit narrowed blue emission band, together with higher photoluminescent quantum yields and reduced radiative lifetime relative to the *mer*‐counterparts. Maximum external quantum efficiencies of 13.5% and 22.8% are achieved for the electrophosphorescent devices based on *f*‐tpb1 and *m*‐tpb1 as dopant emitter together with CIE coordinates of (0.15, 0.23) and (0.22, 0.45), respectively. By using *f‐*tpb1 as the sensitizing phosphor and *t*‐DABNA as thermally activated delayed fluorescence (TADF) terminal emitter, hyperluminescent OLEDs are successfully fabricated, giving high efficiency of 29.6%, full width at half maximum (FWHM) of 30 nm, and CIE coordinates of (0.13, 0.11), confirming the efficient Förster resonance energy transfer (FRET) process.

## Graphical abstract

Blue emissive Ir(III) emitters with functionalized imidazo[4,5‐b]pyridin‐2‐ylidene chelates are reported. One such emitter, namely: *f*‐tpb1, can produce electrophosphorescence with CIE*_x_*_,_ *_y_* = 0.15, 0.23 and maximum EQE of 13.5% and hyperluminescence with CIE*_x_*_,_ *_y_* = 0.13, 0.11 and maximum EQE of 29.6%, respectively.

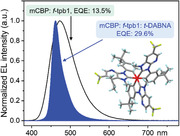

## 1 Introduction

Organic light‐emitting diode (OLED) is an imperative technology to realize full color displays and solid‐state lighting luminaries of the 21st century.^[^ ^1^ ^]^ High‐efficiency blue emitters, no matter whether they were phosphorescent or thermally activated delayed fluorescent (TADF) materials, should have an opportunity for realizing future “blue back panel” concept and enabling new OLED architectures with enlarged panel sizes and lowered manufacturing costs.^[^ ^2^ ^]^ Among these emitters, Ir(III) complexes have dominated the fields of OLED emitters during the past two decades. The Ir(III) metal atom is capable to promote the spin–orbit coupling facilitated intersystem crossing, allowing full utilization of both electro‐excitation generated singlet and triplet excitons.^[^ ^3^ ^]^ The commercially viable Ir(III) phosphors may involve cyclometalating *N*‐heteroaromatic fragment, namely: Ir(C^N)_3_ and Ir(C^N)_2_(L^X), C^N = *N*‐containing aromatics and L^X = anionic ancillary. These molecular designs are particularly suitable for making both red and green emitters; however, due to the inferior ligand‐field strength imposed by their inherent *N*‐donor, unavoidable reduction in efficiency was observed for the blue emitters that demand higher energy emitting states.^[^ ^4^ ^]^

**Scheme 1:**
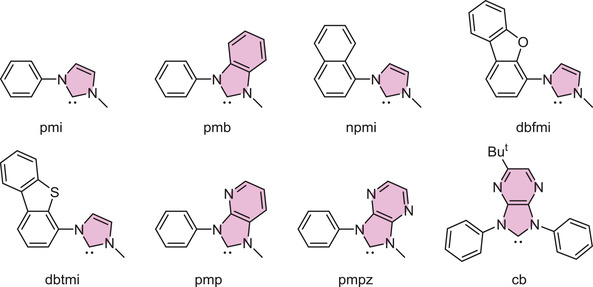
Some representative carbene cyclometalates ligands reported in literature.

One possible way to avoid this stalemate is to replace the neutral N‐donor of C^N chelate with a carbene based fragment in affording the C^C chelate; the latter is known for the stronger metal—ligand bonding, and more destabilized ligand‐centered π*‐orbitals and metal‐centered (MC) dd excited states, all of which are essential in affording efficient blue emission.^[^ ^5^ ^]^ In this regard, Thompson and coworkers reported the first set of Ir(III) carbene complexes for OLEDs.^[^ ^6^ ^]^ Representative C^C chelates involved functional imidazol‐2‐ylidene (pmi)^[^ ^7^ ^]^ and benzimidazol‐2‐ylidene (pmb) fragments,^[^ ^8^ ^]^ to which their drawings are depicted in **Scheme** **1**. However, the inferior purple luminescence of these Ir(III) complexes prohibited the practical industrial applications. In reducing the HOMO/LUMO gap toward true blue, one method is to increase the *π*‐conjugation of carbene chelates. This can be attained by replacement of *N*‐phenyl substituent of carbene cyclometalates with *N*‐naphthalenyl,^[^ ^9^ ^]^ *N*‐dibenzofuranyl,^[^ ^10^ ^]^ and even *N*‐dibenzothiophenyl^[^ ^11^ ^]^ group (*cf*. npmi, dbfmi and dbtmi of Scheme 1). However, these Ir(III) and Pt(II) carbene complexes afforded structured emission bands that were dominated by an enhanced ligand‐centered (LC) ππ* transition contributions, resulting in an undesirable long radiative lifetime for emitters and hence, poor lifespan for OLED devices.^[^ ^12^ ^]^

Alternatively, Thompson and coworkers reported another breakthrough on the Ir(III) carbene emitters with functional imidazo[4,5‐b]pyridin‐2‐ylidene chelate (pmp).^[^ ^13^ ^]^ These Ir(III) carbene complexes have given true blue electroluminescence with CIE coordinates of (0.16, 0.09) and very high brightness (>7800 cd m^−2^), after incorporation of the so‐called graded doping technology in emissive layer (EML). Relevant homoleptic,^[^ ^14^ ^]^ heteroleptic,^[^ ^15^ ^]^ and even derivatives with tridentate carbene pincer chelate^[^ ^16^ ^]^ were also tested, with attempts in affording efficient blue emission, or even luminescence across the whole visible region. Eventually, homoleptic Ir(III) carbene complexes with imidazo[4,5‐b]pyrazin‐2‐ylidene donor groups (e.g., pmpz) or with 7,9‐dihydro‐8H‐purin‐8‐ylidene chelates were proved to be better blue phosphors.^[^ ^17^ ^]^ Despite having high OLED performances, however, synthesis of these imidazo[4,5‐b]pyrazin‐2‐ylidene chelates suffered from unintended methylation at the N atom of the pyrazine unit,^[^ ^18^ ^]^ while the lack of coordination selectivity for chelate cb also caused formation of an inseparable mixture of configurational isomers for Ir(cb)_3_.^[^ ^17a^ ^]^ All of these problems must be solved for “pyrazine” functionalized chelates before they can find practical applications.

**Scheme 2:**
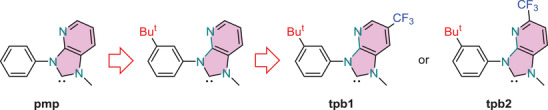
A synthetic transformation that reduced the ππ* gap of carbene chelates.

**Scheme 3:**
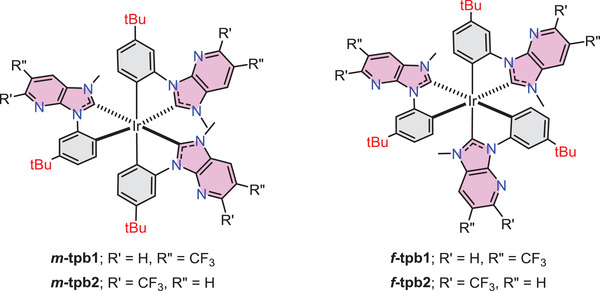
Structural drawings of Ir(III) complexes with CF_3_‐substituted imidazo[4,5‐b]pyridin‐2‐ylidene chelates.

Herein, we reported a promising method for reducing the HOMO/LUMO energy gap of Ir(III) phosphors based on the parent pmp chelate. As depicted in **Scheme** **2**, the new designs possess an electron donating *t*‐butyl substituent on the *N*‐phenyl appendage, and an electron‐withdrawing CF_3_ substituent on the peripheral imidazo[4,5‐b]pyridin‐2‐ylidene unit. Four homoleptic Ir(III) complexes *m*‐tpb1 and *m*‐tpb2 and *f*‐tpb1 and *f*‐tpb2, were synthesized from the tailor‐made chelates, and their drawings are depicted in **Scheme** **3**. Most excitingly, all of them possessed narrowband width (for the *f*‐isomers), high photoluminescence quantum yield, and relatively shortened radiative lifetime as recorded in degassed toluene solution at RT. In addition, bright and highly efficient electrophosphorescence were successfully achieved using *f*‐tpb1 and *m*‐tpb1 as dopant emitters, giving maximum external quantum efficiencies (EQEs) of 13.5% and 22.8%, respectively. Importantly, we then applied *f*‐tpb1 as sensitizer and 12‐di‐*tert*‐butyl‐5,9‐bis(4‐(*tert*‐butyl)phenyl)‐5,9‐dihydro‐5,9‐diaza‐13b‐boranaphtho[3,2,1‐de]anthracene (*t*‐DABNA) as TADF terminal emitter.^[^ ^19^ ^]^ As a result, efficient hyperluminescent OLED devices^[^ ^2^ ^]^ were fabricated, rendering much desired deep‐blue luminescence with maximum EQE as high as 29.6%, accompanied by a narrow full width at half maximum (FWHM) of 30 nm and Commission Internationale de L'Eclairage (CIE) coordinates of (0.13, 0.11).

## 2 Results and Discussion

### 2.1 Syntheses and Characterization

The carbene pro‐chelates were prepared according to the protocols depicted in Scheme S1, Supporting Information. The key starting materials were 2‐chloro‐3‐nitro‐5‐(trifluoromethyl)pyridine (A1) and 2‐chloro‐3‐nitro‐6‐(trifluoromethyl)pyridine (B1), to which the latter was prepared from commercially available 2‐chloro‐6‐(trifluoromethyl)‐3‐pyridinamine by oxidation of amine with a solution of H_2_O_2_ and H_2_SO_4_.^[^ ^20^ ^]^ After that, the demanded imidazo[4,5‐b]pyridin‐1‐ium pro‐chelates (A5 and B5) were prepared, followed by the multi‐step syntheses involving i) chloride‐to‐aniline substitution, ii) nitro‐to‐amine reduction, iii) formic acid cyclization and, finally, iv) addition of methyl trifluoromethanesulfonate (CF_3_SO_3_Me). These carbene chelates were next treated with *mer*‐trichloridotris(tetrahydrothiophene‐*κ*S)iridium(III), *mer*‐IrCl_3_(THT)_3_, in the presence of sodium acetate in refluxing *tert*‐butylbenzene solution. For both reactions, *m*‐isomers *m*‐tpb1 and *m*‐tpb2, and *f*‐isomers *f*‐tpb1 and *f*‐tpb2, were obtained as the major and minor products, respectively. Moreover, the *m*‐isomers could be easily converted to the *f*‐isomers using trifluoroacetic acid as the catalyst.^[^ ^21^ ^]^ Hence, both homoleptic isomers could be prepared in reasonable quantity for subsequent investigations.

**Figure 1:**
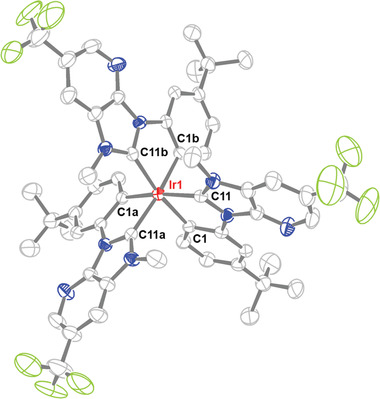
Structure drawing of *f*‐tpb1 with thermal ellipsoids shown at 30% probability level. Selected bond length (Å): Ir1‐C11 = 2.028(2) and Ir1‐C1 = 2.088(2). Selected bond angle (°): *trans*‐C11‐Ir1‐C1a = 168.63(9), *cis*‐C11‐Ir1‐C1 = 77.99(9), C1‐Ir1‐C1a = 90.65(9), and C11‐Ir1‐C11a = 102.07(8).

As a proof of structure, single crystal X‐ray structural diffraction study on *f*‐tpb1 was executed. **Figure** **1** depicts its structural diagram, to which the molecule adopts a slightly distorted octahedral geometry, with carbene Ir‐C distances (2.028(2) Å) being notably shorter than those of phenyl cyclometalates (2.088(2) Å), confirming the common metric parameters that have been observed in many Ir(III) complexes bearing cyclometalating carbene chelate(s).^[^ ^6^, ^22^ ^]^

### 2.2 Photophysical Measurement

**Figure 2:**
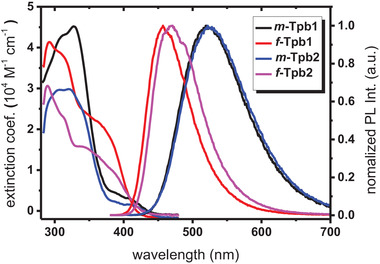
Photoluminescence spectra and lifetimes of the studied homoleptic Ir(III) carbene complexes measured in degassed toluene at RT (10^−5^ m).

**Figure** **2** shows the UV–vis absorption and emission spectra of the studied complexes measured in toluene at RT. **Table** **1** summarizes the corresponding spectral and exciton dynamic data. As can be seen, absorption bands with wavelengths greater than 350 nm indicate ligand‐centered ππ* transitions as well as possible contribution from the inter‐ligand charge transfer transitions. The lower energy absorption bands in the region starting from ≈ 360 nm are mainly attributed to the spin‐allowed (singlet) metal‐to‐ligand charge transfer (MLCT) transition, and in part with ligand‐to‐ligand charge transfer (LLCT) transition. It is notable that both *m*‐isomers exhibit relatively lower absorption extinction coefficient than that of *f*‐isomers, but with absorption onset extended beyond ≈460 nm (cf. ≈450 nm in *f*‐isomers). This distinction could be attributed to different geometrical arrangements, where *m*‐isomers possess three distinct cyclometalating carbene chelates, resulting in broadened MLCT transition, and lowered absorption extinction coefficient and longer spectral onset. In contrast, the symmetrical arrangement of carbene chelates of *f*‐isomers provided a narrow absorption band with higher absorption coefficient.

**Table 1:** Photophysical data of the studied Ir(III) complexes in solution

	abs *λ*_max_ [nm] [*ε* × 10^4^ m ^−1^·cm^−1^]^a)^	PL *λ*_max_ [nm]^a)^	FWHM [cm^−1^ nm^−1^]^b)^	*Φ*_P_ [%]^a,^ ^c)^	*τ*_obs_ [µs] ^a)^	*τ*_rad_ [µs]^d)^	*k*_r_ [10^6^ s^−1^]^e)^	*k*_nr_ [10^5^ s^−1^]^f)^
*m*‐tpb1	309 (4.3), 328 (4.5), 406 (0.3)	520	3856/108	69	0.83	1.20	0.83	0.37
*f*‐tpb1	292 (4.1), 313 (3.8), 361 (2.2)	457	3228/70	85	0.76	0.90	1.11	0.20
*m*‐tpb2	307 (3.0), 320 (3.0), 405 (0.2)	522	3913/111	65	0.87	1.34	0.75	0.40
*f*‐tpb2	290 (3.1), 314 (2.3), 336 (1.6)	470	3511/80	71	0.81	1.13	0.88	0.36

^a)^ Measured in degassed 1 × 10^−5^ m toluene at RT;

^b)^ FWHM: full width at half maxima of PL emission peak max. in cm^−1^;

^c)^ Quantum yields measured using Coumarin 102 (C102) in methanol (Q.Y. = 80% and *λ*max = 480 nm) as standard;

^d)^ *τ*_rad_ = *τ*_obs_/*Φ*_P_;

^e)^ k_r_ = *Φ*_P_/*τ*_obs_;

^f)^ k_nr_ = (1‒*Φ*_P_)/*τ*_obs_

Emissions were next measured in degassed toluene solution at RT. As depicted in Figure 2, the *m*‐isomers exhibit structureless emission profile with onset at ≈450 nm and peak wavelength at 520 and 522 nm, respectively. In contrast, the corresponding *f*‐isomers show a much blue‐shifted emission onset at ≈400 nm, with peak wavelength at 457 and 470 nm, and a significantly reduced FWHM of 70 and 80 nm in comparison to their *m*‐isomers (FWHM = 108 and 111 nm). The greater red shift in emission wavelengths and FWHM of *m*‐isomers are due to the dissymmetrical nature of the associated carbene chelates. The multiple MLCT transition to each individual carbene chelate, together with the associated LLCT transition, induces more solvent relaxation in solution state, that is, the solvatochromism. Support of this viewpoint is revealed in the photophysical data taken in PMMA polymer matrix (Figure S5, Supporting Information), where the emission peak max. at 490 and 492 nm were observed for *m*‐tpb1 and *m*‐tpb2, which are blue shifted by as large as 30 nm compared to that (520 and 522 nm, respectively) in toluene. Oppositely, as shown in Table S1, Supporting Information, the emission in PMMA polymer is red shifted as small as 3 and 9 nm (cf. in toluene) for *f*‐tpb1 and *f*‐tpb2, respectively, affirming our proposition.

Moreover, despite having higher emission energy, both isomers *f*‐tpb1 and *f*‐tpb2 exhibit better photoluminescent quantum yields (PLQYs) of 85% and 71% than that of *m*‐tpb1 (69%) and *m*‐tpb2 (65%) in degassed toluene at RT, while *f*‐tpb1 and *f*‐tpb2 display slightly smaller observed emission lifetimes of 0.76 and 0.81 µs, respectively. Their radiative lifetime, which is derived from the observed lifetime divided by PLQY, is calculated to be 0.90 and 1.13 µs (*cf*. Table 1). Notably, these radiative lifetimes are also found to be shorter than their *m*‐counterparts *m*‐tpb1 (1.20 µs) and *m*‐tpb2 (1.34 µs), manifesting a greater degree of spin–orbit coupling and oscillator strength for *f*‐tpb1 and *f*‐tpb2, which could be more advantageous for fabrication of efficient OLED devices with suppressed efficiency roll‐off.

Electrochemical properties and thermal stabilities. Electrochemical data of these Ir(III) complexes were examined using cyclic voltammetry conducted in acetonitrile. All complexes showed nearly undetectable reduction process and, hence, only the oxidation potentials were reported, shown in **Table** **2**; Figure S4, Supporting Information. As expected, these processes were all associated with the metal‐centered oxidation, while the quasi‐reversible half‐wave oxidation potential for *m*‐tpb1 and *m*‐tpb2 occurred at 0.47 and 0.47 V, among which the *f*‐isomers exhibited an anodic shift of 0.20 and 0.23 V in comparison to their *m*‐counterparts. Using their onset potentials, HOMO energy levels of −5.22 (*m*‐tpb1), −5.46 (*f*‐tpb1), −5.23 (*m*‐tpb2), and −5.44 V (*f*‐tpb2) were calculated and depicted in Table 2. Concurrently, their respective LUMO energy levels can also be estimated using the equation: *E*_LUMO_ = *E*_HOMO_ – *E*_g_; *E*_g_ = absorption energy gap.

**Table 2:** Summarized electrochemical data and energy gap of the studied Ir(III) complexes

Complex	*E*ox ½ (*ΔE*_p_)^a)^ [V]	*E*onset ox [V]	*E*_HOMO_ [eV]^b)^	*E*_g_ [eV]	*E*_LUMO_ [eV]
*m*‐tpb1	0.47 (0.07)	0.41	−5.22	2.80	−2.42
*f*‐tpb1	0.67 (0.06)	0.65	−5.46	3.02	−2.44
*m*‐tpb2	0.47 (0.08)	0.42	−5.23	2.78	−2.55
*f*‐tpb2	0.70 (0.06)	0.63	−5.44	2.98	−2.46

^a)^ Data measured in MeCN solution, E_1/2_ = [(*E*_pa_ + *E*_pc_)/2], where ox and re are oxidation and reduction potential, and *E*_pa_ and *E*_pc_ are the anodic and cathodic wave potential, respectively, referenced to Ag/AgCl electrode (pre‐calibrated with ferrocene redox couple Fc+/Fc [0.31 V]);

^b)^ FMO energy levels are calculated from the equations: E_HOMO_ = −(*E*_ox_^onset^−E_Fc/Fc_+ + 4.8) eV. Energy gap *E*_g_ is estimated from the emission onset using equation *E*_g_ = 1240/*λ*_PL onset_.

### 2.3 Theoretical Investigation

The lowest singlet (S_1_) and lowest triplet (T_1_) excited states for the studied Ir(III) carbene complexes were investigated by TD–DFT calculations^[^ ^23^ ^]^ to gain understanding into the nature of the absorption/emission bands and the effect of chelate modification on their photophysical properties. Details of computational methods are described in the Experimental Section, Supporting Information.

**Figure 3:**
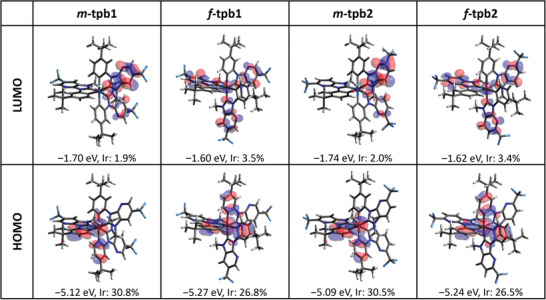
Frontier molecular orbitals (HOMO and LUMO) of the studied Ir(III) complexes at their geometries optimized for the ground state. The orbital energy level and the contribution from Ir atom are also provided. Note the HOMO to LUMO orbital transition contributes dominantly to the T_1_ excited state for all four complexes (*cf*. Table 3).

The main calculation results are shown in **Table** **3** and **Figure** **3**. The calculated energies of the S_1_ state in terms of wavelength are 443, 402, 443, and 411 nm for *m*‐tpb1, *f*‐tpb1, *m*‐tpb2, and *f*‐tpb2, respectively (*cf*. Table 3), consistent with their experimental lowest‐energy absorption tail at 450–460 nm (*cf*. Figure 2). The calculated energies of T_1_ excited state are 452, 425, 452, and 433 nm for *m*‐tpb1, *f*‐tpb1, *m*‐tpb2, and *f*‐tpb2, respectively (*cf*. Table 3), showing the same trend versus experimental phosphorescence (peaking at 520, 457, 522, and 470 nm, respectivel*y* [Figure 2 and Table 1]. The results reveal two trends: i) structural change from a meridional isomer to its facial isomer leads to a blue‐shifted emission (by 0.11–0.17 eV); ii) whereas the change in the position of the CF_3_ substituent at the imidazo[4,5‐b]pyridin‐2‐ylidene chelates (*cf*. tpb1 → tpb2) affects little in the emission energy (within 0.06 eV). Note the deviation between calculated T_1_ energy and the experimental emission wavelength is ≈0.3 eV (6.9 kcal mol^−1^). This deviation is common for cyclometalated Ir(III) complexes, which is mainly caused by the uncertainties of both the applied theoretical levels and adopted geometrical structure of the emission states. Nevertheless, the trend of the energy gap obtained by the theoretical approach is consistent with that of the experimental result. The analysis of calculated T_1_ excited state would allow us to gain a certain level of understanding in phosphorescence property elaborated below.

**Table 3:** The calculated excitation energy (*λ*), oscillator strength (*f*), and main orbital contributions of the lowest singlet (S_1_) and triplet (T_1_) excited states, and the assignment of charge characters of the T_1_ state for the studied Ir(III) complexes at their geometries optimized for the ground state

	State^a)^	*λ* [nm/eV]	*f*	Orbital contribution (>10%)	Assignment^b)^
*m*‐tpb1	T_1_	452/2.75	0	HOMO → LUMO (87%)	MLCT (25.1%), ILCT, LLCT
	S_1_	443/2.80	0.0063	HOMO → LUMO (96%)	
*f*‐tpb1	T_1_	425/2.92	0	HOMO → LUMO (81%)	MLCT (18.9%), ILCT
	S_1_	402/3.08	0.0602	HOMO → LUMO+1 (92%)	
*m*‐tpb2	T_1_	452/2.75	0	HOMO → LUMO (87%)	MLCT (24.8%), ILCT, LLCT
	S_1_	443/2.80	0.0049	HOMO → LUMO (96%)	
*f*‐tpb2	T_1_	433/2.86	0	HOMO → LUMO (79%)	MLCT (18.3%), ILCT
	S_1_	411/3.02	0.0444	HOMO → LUMO+1 (91%)	

^a)^ The results were calculated by TD–DFT using B3LYP functional with PCM for modeling the toluene solvent (see computational details in the Supporting Information for more information);

^b)^ The percentage of MLCT character of T_1_ state was calculated from the change in the metal contribution in the dominant electronic transition (cf. Figure 3) multiplied with its contribution weight.

The calculation results (*cf*. Table 3) indicate that the T_1_ state is contributed dominantly (≥79%) by the HOMO to LUMO transition for all four Ir(III) complexes. Therefore, the analysis of the localization and orbital energy of frontier orbitals is helpful for understanding the charge characters of the T_1_ state and how the ligand modification affects the energy of the T_1_ state and thus the emission energy. The HOMO for all four complexes is mainly localized at the Ir(III) metal atom and the 3‐*tert*‐butylphenyl cyclometalates (two of three fragments for *m*‐isomers and all three for *f*‐isomers), while the LUMO is mainly localized at two (for *m*‐isomers) or three (for *f*‐isomers) of imidazo[4,5‐b]pyridin‐2‐ylidene fragments. Therefore, the T_1_ state of both *m*‐isomers shows a mixture of metal‐to‐ligand charge transfer (MLCT) intra‐ligand charge transfer (ILCT) and ligand‐to‐ligand charge transfer (LLCT) characters, whereas that of both *f*‐isomers shows a mixture of MLCT and ILCT characters. The structural change from *m*‐isomers to *f*‐isomers stabilized the HOMO and meanwhile destabilized the LUMO, thus leading to a blue‐shifted emission.

The effect of ligand modification on the radiative rate (*k*_r_) may be understood from the analysis of the percentage of MLCT transition in the T_1_ state and the localization of the pair of orbitals dominating the T_1_ state. As discussed in our previous research, the interplay of these two components largely determines the magnitude of radiative rate constant of the T_1_ state.^[^ ^20^ ^]^ On the one hand, more MLCT percentage involved in the transition should give the greater radiative rate constant to the T_1_ state due to the direct involvement of metal atom, facilitating intersystem crossing.^[^ ^24^ ^]^ On the other hand, the increasing orbital overlap (between the dominant pair of orbitals contributing to the T_1_ state) also results in an increased radiative rate constant due to an increased electron delocalization and hence, the transition dipole moment.^[^ ^20^ ^]^ The experimental results indicate that the *f*‐isomers achieved a greater radiative rate constant than that of the relevant *m*‐isomers (the same trend as the calculated *k*_r_, *cf*. Table S1, Supporting Information). This might be rationalized by an increased orbital overlap between the HOMO and LUMO in the *f*‐isomers (*cf*. Figure 3), although there is a small reduction in their MLCT character of their T_1_ state.

### 2.4 Device Fabrication and Characterization

**Figure 4:**
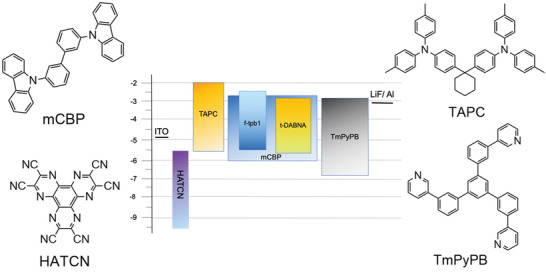
Device structure based on *f*‐tpb1 and *m*‐tpb1 phosphors and molecular structures of organic functional materials.

Two Ir(III) complexes, *f*‐tpb1 and *m*‐tpb1 were selected to investigate their electroluminescence properties due to their better photophysical properties. Considering the triplet energy levels of these two materials are lower than 2.9 eV, 3,3’‐di(9*H*‐carbazol‐9‐yl)‐1,1’‐biphenyl (mCBP) was selected as the host material due to its relatively higher triplet energy level and good charge transporting capability.^[^ ^19^ ^]^ Multilayer electrophosphorescent devices were then fabricated with a device architecture: indium tin oxide (ITO)/ 1,4,5,8,9,11‐hexaazatriphenylene hexacarbonitrile (HATCN, 10 nm)/ 1,1‐bis[(di‐4‐tolylamino)phenyl] cyclohexane (TAPC, 25 nm)/ mCBP: *X* wt% phosphors (30 nm)/ 1,3,5‐tri(*m*‐pyridin‐3‐ylphenyl)benzene (TmPyPB, 35 nm)/ lithium fluoride (LiF, 1 nm)/ Al (100 nm). The energy diagram and chemical structures of the organic layers are shown in **Figure** **4**.

**Figure 5:**
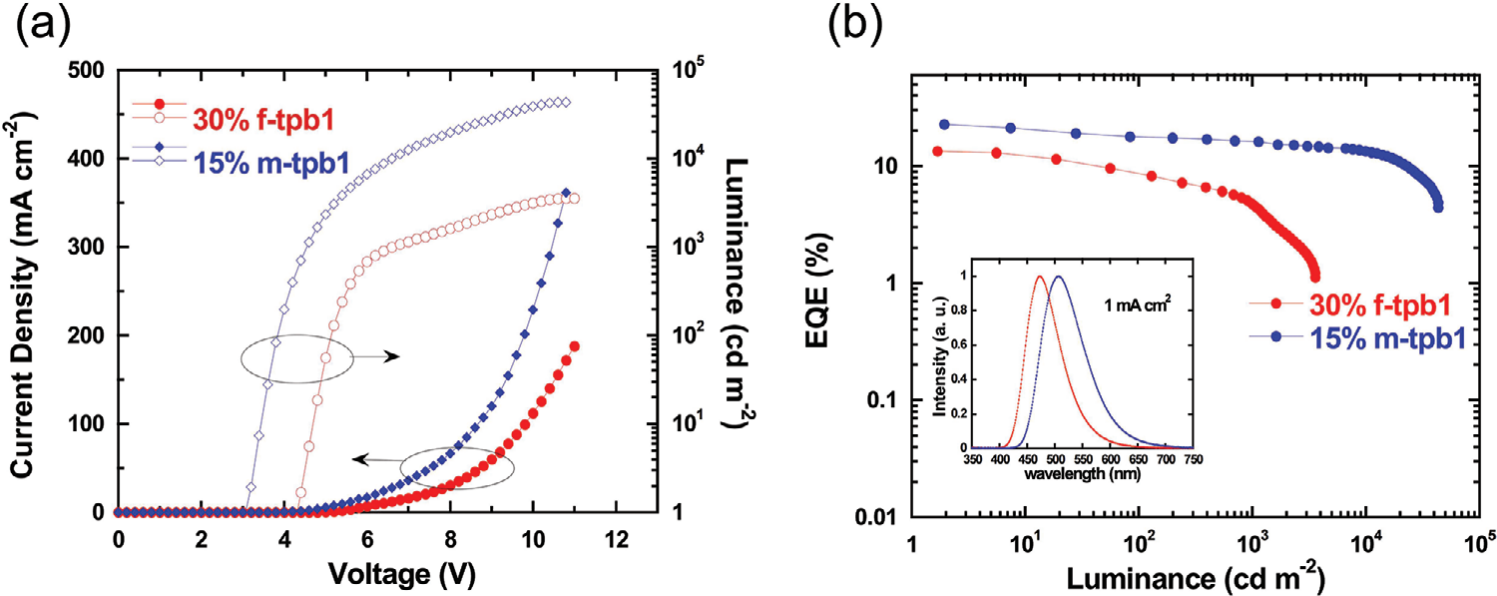
Device performance based on *f*‐tpb1 and *m*‐tpb1 as phosphorescent emitter with optimized doping ratios (30% for *f*‐tpb1 in mCBP and 15% for *m*‐tpb1). a) Current density–voltage–luminance characteristics; b) EQE to current density curve with EL spectra inserted.

First, the OLED devices based on *f*‐tpb1 and *m*‐tpb1 as emitters are fabricated with doping concentrations of 7, 10, and 15 wt%. As shown in Figures S8 and S9, Supporting Information, the turn‐on voltage (*V*_on_) of the devices based on *f*‐tpb1 increased with the increasing doping concentration, which suggests an inferior carrier transport capability of *f*‐tpb1. In contrast, *m*‐tpb1 with better carrier transport capability shows higher current density and the *V*_on_ decreased with the increasing doping concentration.^[^ ^19^, ^25^ ^]^ As illustrated in Figures S8b and S9b, Supporting Information, both EQEs and maximum brightness (*L*_max_) of the devices based on *f*‐tpb1 and *m*‐tpb1 increased with increasing doping concentration. Further improved EQE can be achieved for the device based on *f*‐tpb1 when its doping concentration was increased to ever higher ratios (i.e., 20, 30, and 40 wt%). As shown in Figure S10, Supporting Information, although high efficiency of 14.9% was achieved for the device based on *f*‐tpb1 with a high doping concentration of 40 wt%, it shows slightly red‐shifted electroluminescence (EL) spectrum compared with those devices with lower doping concentrations (*cf*. Figure S10 and Table S3, Supporting Information). As a result, the doping concentration of *f*‐tpb1 was optimized to be 30 wt% for a fair comparison, and the EL spectra and EQE versus luminance characteristics of the devices based on *f*‐tpb1 and *m*‐tpb1 are shown in **Figure** **5**. The maximum EQEs were recorded to be 13.5% and 22.8% for the devices based on *f*‐tpb1 and *m*‐tpb1, respectively. It is also of interest that the efficiency of *m*‐tpb1 retains over 10% even at the luminance of 22 960 cd m^−2^, indicating a significantly suppressed efficiency roll‐off. All the devices show solely emission peak of phosphors free from any interfering emission by the host and adjacent layers, indicating that the excitons can be finely restricted in the emitting layer and the excitonic energy can be efficiently transferred to the phosphorescent dopant. The EL emission of *f*‐tpb1 showed an emission peak of 472 nm with CIE chromaticity coordinates of (0.15, 0.23), which located in the blue spectroscopic region. While *m*‐tpb1 exhibited green emission with an EL peak at 503 nm and bluish‐green CIE coordinates of (0.21, 0.45).

**Figure 6:**
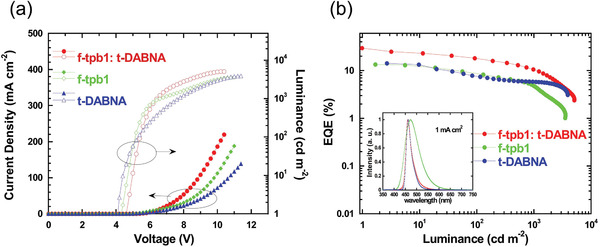
The performance of hyperphosphorescent devices using mCBP: 30% *f*‐tpb1: 1% *t*‐DABNA as emitting layer (EML) and the corresponding phosphorescent and TADF devices using mCBP: 30 wt% *f*‐tpb1 and mCBP: 1 wt% *t*‐DABNA as EML. a) Current density–voltage–luminance characteristics; b) EQE to current–density curve with EL spectra inserted.

With the aim of realizing deep‐blue OLEDs, *f*‐tpb1 was further selected to sensitize TADF terminal emitter *t*‐DABNA, because *t*‐DABNA shows a deep blue emission with narrow‐band FWHM and the emission of *f*‐tpb1 has a large spectral overlap with the absorption of *t*‐DABNA and related BN‐based MR TADF emitters.^[^ ^19^, ^25^, ^26^ ^]^ The employed device architecture was: ITO/ HATCN (10 nm)/ TAPC (25 nm)/ mCBP: *X*% phosphor: *Y*% *t*‐DABNA (30 nm)/ TmPyPB (35 nm)/ LiF (1 nm)/ Al (100 nm) (*cf*. Figure S11). Herein, the concentration of *f*‐tpb1 was set to be 30 wt% and the concentration of *t‐*DABNA was tuned from 0.5 to 2 wt%. The device performances were summarized in **Table** **4**; Table S5, Supporting Information. As shown in Table S5 and Figure S12, Supporting Information, maximum current efficiency (CE) was recorded to be 28.7 cd A^−1^ at the *t*‐DABNA doping ratio of 1 wt%, corresponding to a maximum EQE of 29.6%. To the best of our knowledge, this is the highest EQE for the hyperluminescent device with *t*‐DABNA as the TADF terminal emitter (*cf*. Table S6, Supporting Information). The hyperluminescence based on *f*‐tpb1 show an emission peak wavelength at 462 nm and FWHM of 30 nm, which are identical to those of the non‐sensitized TADF devices with *t*‐DABNA as emitter, indicating an efficient FRET process from *f*‐tpb1 to *t*‐DABNA. In addition to the narrow‐band blue emission, the maximum EQE of the sensitized device is also significantly higher than that of the non‐sensitized one, suggesting its higher exciton utilization (**Figure** **6**b). For the device based on *t*‐DABNA without the phosphor sensitizer, the reverse intersystem crossing (RISC) is slow due to its large energy difference between triplet and singlet energy level (*ΔE*_ST_), resulting in the accumulation and quenching of triplet excitons, which restricted the exciton utilization.^[^ ^27^ ^]^ In contrast, within this hyperluminescent device, triplet excitons are mostly generated on *f*‐tpb1 and then transferred to the singlet excited state of *t*‐DABNA through efficient FRET process. The fast radiative transition of the singlet excitons of *t*‐DABNA greatly improved exciton utilization and thus greatly improved device performance.^[^ ^28^ ^]^

**Table 4:** Summarized EL properties of electrophosphorescent, hyperluminescent, and TADF devices

EML	*V*_on_ ^a)^ [V]	*L*_max_ [cd m^−2^]	Max. EQE / CE / PE [% / cd A^−1^ / lm W^−1^]	*λ*_max_ [nm]	FWHM^b)^ [nm]	CIE [*x*,*y*]
mCBP: 30% *f*‐tpb1	4.4	3558	13.5 / 22.8 / 16.3	472	73	0.15, 0.23
mCBP: 15% *m*‐tpb1	3.2	43 261	22.8 / 61.7 / 60.6	503	88	0.22, 0.45
mCBP: 30% *f*‐tpb1: 1% *t*‐DABNA	4.6	5084	29.6 / 28.7 / 19.6	462	30	0.13, 0.11
mCBP: 1% *t*‐DABNA	4.1	3886	14.2 / 12.8 / 9.6	463	26	0.14, 0.10

^a)^ Data recorded at 1 cd m^−2^;

^b)^ Full width at half maximum of EL spectrum.

The energy transfer and emission processes were further investigated by transient PL analysis of three EML thin films of i) mCBP: *f*‐tpb1, ii) mCBP: *t*‐DABNA, and iii) mCBP: *f*‐tpb1: *t*‐DABNA (*cf*. Figure S13 and Table S7, Supporting Information). As shown in Table S7, Supporting Information, the exciton lifetime of mCBP: 30% *f*‐tpb1 was estimated to be 1.8 µs, which is the excited state lifetime of *f*‐tpb1 through phosphorescence. The mCBP: 1% *t*‐DABNA film shows a prompt lifetime of 7.1 ns and a long delayed lifetime of 171.4 µs, which are attributed to the prompt fluorescence transition and TADF transition, respectively. The ternary co‐doped film of mCBP: 30% *f*‐tpb1: 1% *t*‐DABNA shows a relatively complex decay, which consists of a prompt lifetime of 12.1 ns and delayed lifetimes of 0.5 and 94.9 µs. The prompt lifetime can be attributed to the energy transfer from the singlet exciton of the host to the singlet exciton of *t*‐DABNA and thus, radiative transition. The delayed lifetime of 0.5 µs can be attributed to the FRET from the triplet exciton of *f*‐tpb1 to the singlet exciton of *t*‐DABNA, followed by irradiation. The second delayed lifetime of 94.9 µs originates from the inevitable Dexter energy transfer from the host to *t*‐DABNA and thus the reversed intersystem crossing (RISC) and radiative transition of *t*‐DABNA. The relatively shorter delayed lifetimes of the mCBP: 30% *f*‐tpb1: 1% *t*‐DABNA film compared with the mCBP: 1% *t*‐DABNA film indicate the efficient energy transfer in giving hyperluminescence. Owing to the fast triplet exciton conversion in the mCBP: 30% *f*‐tpb1: 1% *t*‐DABNA film, the accumulation and quenching of triplet excitons will be significantly reduced to give higher exciton utilization and reduced efficiency roll‐off. These results thus suggest that imidazo[4,5‐b]pyridin‐2‐ylidene chelate can be employed in synthesizing Ir(III) phosphors for generation of both the blue electrophosphorescence and hyperluminescence. Despite the efficient energy transfer, the direct carrier trap by the TADF terminal emitter cannot be omitted in the electrical excitation process, which may lead to long delayed lifetime according to the slow RISC and thus, potential exciton annihilation.

We like to point out that the hyperluminescence described in the present article belongs to the class of phosphor sensitized fluorescent OLED first proposed by Baldo et.al.^[^ ^29^ ^]^ However, due to the unavailability of efficient blue phosphor and fluorophore at that time, the associated proof‐of‐the‐concept study was only focused on the green phosphor and red fluorophore. Next, this seminar work by Baldo triggered many more studies on both the phosphor sensitized fluorescent^[^ ^30^ ^]^ and phosphor sensitized TADF devices.^[^ ^19^, ^25^, ^26^ ^]^ More recently, TADF sensitizers have been actively tackled in obtaining TADF assisted fluorescent OLED^[^ ^31^ ^]^ and TADF OLED,^[^ ^32^ ^]^ particularly to the blue emissive OLED devices. Therefore, the new term of hyperluminescence (or hyperphosphorescence)^[^ ^2^ ^]^ was raised to provide a clear distinction from traditional hyperfluorescence^[^ ^33^ ^]^ in highlighting the vest potential of phosphor sensitized TADF OLED devices, for its better utilization of triplet excitons and more suitable in fabrication of efficient and long lifespan (blue emissive) hyper‐OLED devices.

## 3 Conclusion

In summary, we designed and synthesized four homoleptic iridium(III) complexes possessing CF_3_‐substituted imidazo[4,5‐b]pyridin‐2‐ylidene cyclometalating fragments. All prepared homoleptic iridium(III) complexes, that is, *f*‐tpb1, *f*‐tpb2, *m*‐tpb1, and *m*‐tpb2, exhibited high PL quantum yields in the range of 65–85%, narrow‐band blue emission for *f*‐isomers, and shortened radiative lifetimes in degassed toluene solution at RT. As a proof‐of‐the‐concept, *f*‐tpb1 and *m*‐tpb1 were investigated as the dopants to generate efficient electrophosphorescence. High efficiencies of 13.4% and 22.8% were achieved for the *f*‐tpb1 and *m*‐tpb1 based OLED devices, with blue and bluish‐green emissions peaking at 472 and 503 nm corresponding to CIE coordinates of (0.15, 0.23) and (0.22, 0.45), respectively. Furthermore, hyperluminescence using *f*‐tpb1 as assistant sensitizer and *t*‐DABNA as terminal emitter was also successfully developed, giving an unprecedented high EQE of 29.6% and a narrow‐band deep blue emission with CIE coordinates of (0.13, 0.11) and FWHM of 30 nm, respectively. These results demonstrate that the CF_3_‐substituted imidazo[4,5‐b]pyridin‐2‐ylidene‐based iridium(III) complexes can be employed for generation of both blue emissive electrophosphorescence and hyperluminescence.

## 4 Experimental Section

### General Information and Materials

Commercially available reagents were used without further purification. ^1^H and ^19^F NMR spectra were measured with 400 MHz NMR instrument (Bruker AVANCE III, BBO probe). Elemental analysis was carried out on an Elemental Micro Carbon‐Hydrogen–Nitrogen Analyzer (Elementar VARIO Micro Cube). Mass spectra were recorded on an Applied Biosystems 4800 Plus MALDI TOF/TOF Analyzer using 2,5‐dihydroxybenzoic acid as the matrix substance.

### Photophysical Measurements

UV–vis absorption spectra were measured with a UV–visible NIR spectrophotometer system (HITACHI U‐3310). Steady‐state emission spectra were measured with a spectrofluorometer (Edinburgh FLS920) and lifetime decay profiles were measured with a time‐correlated single photon counting (TCSPC) system coupled with a mode‐locked Ti:Sapphire laser (Spectra Physics, Model 3960) followed by a pulse picker (Spectra Physics, Model 3980). The picked pulse then passed through a frequency‐doubling crystal (BBO) to generate the excitation source. Last, the polarizer between the sample chamber and detector was set at magic angle relative to the excitation source in order to eliminate the polarization effects. All solution samples were degassed using at least three freeze‐pump‐thaw cycles. Photoluminescence quantum yields in solution at RT were calculated using Coumarin 102 (C102) in methanol (Q.Y. = 0.87) as the standard with corrections on refractive indices of different solvents, while quantum yields in PMMA thin film were measured by an integrated sphere. Lifetimes for thin films were performed by an Edinburgh FLS980 time‐correlated single photon counting (TCSPC) system with an EPL‐375 diode laser as the excitation source.

### Electrochemistry

Cyclic voltammetry was measured with an electrochemical analyzer (CHI660) equipped with a three‐electrode system (glassy carbon: working electrode, platinum wire: auxiliary electrode, Ag/AgCl: reference electrode). Nitrogen‐purged acetonitrile was used as solvent and NBu_4_PF_6_ (0.1 m) was used as supporting electrolyte. The potentials were referenced externally to the ferrocenium/ferrocene (Fc^+^/Fc) couple.

Synthesis of *mer*‐ and *fac*‐[tris(3‐(3‐*tert*‐butylphenyl)‐1‐methyl‐5‐(trifluoromethyl)‐3*H*‐1*λ* ^4^‐imidazo[4,5‐b]pyridine‐*C,C^2′^*) iridium(III)] (*m*‐tpb1 and *f*‐tpb1)

A mixture of A5 (1.45 g, 3.0 mmol), *mer*‐trichloridotris(tetrahydrothiophene‐*κ*S)iridium(III) (*mer*‐IrCl_3_(THT)_3_) (563 mg, 1 mmol) and NaOAc (8.2 g, 10 mmol) in 20 mL of *tert*‐butylbenzene was refluxed at 170 °C for 20 h to give a light‐yellow suspension. After cooling to RT, the mixture was filtered through Celite. The solution was concentrated, and the residue dissolved in ethyl acetate and washed with distilled water (100 mL). The organic layer was separated and concentrated to dryness. The crude product was further purified via silica gel column chromatography using hexane/ethyl acetate (5/1, v/v) as the eluent to afford a pale‐green solid m‐tpb1; yield: 503 mg, 42.0% and an off‐white solid f‐tpb1; yield: 347 mg, 29.0%.

Spectral data of *m*‐tpb1: ^1^H NMR (400 MHz, CDCl_3_) *δ* 8.98 (d, *J* = 1.8 Hz, 1H), 8.95 (s, 1H), 8.90 (d, *J* = 1.8 Hz, 1H), 8.78 (s, 1H), 8.77 (s, 1H), 8.75 (s, 1H), 7.74−7.69 (m, 3H), 6.89 (dd, *J* = 7.8, 1.8 Hz, 1H), 6.81 (dd, *J* = 7.7, 1.7 Hz, 1H), 6.80 (s, 2H), 6.75 (d, *J* = 7.7 Hz, 1H), 6.49 (d, *J* = 7.7 Hz, 1H), 3.39 (s, 3H), 3.33 (s, 3H), 3.25 (s, 3H), 1.38 (s, 9H), 1.36 (s, 9H), 1.35 (s, 9H). ^19^F NMR (376 MHz, CDCl_3_) *δ* ‐60.28 (s, 3F), ‐60.35 (s, 3F), −60.40 (s, 3F).

Spectral data of *f*‐tpb1: ^1^H NMR (400 MHz, CDCl_3_) *δ* 8.92 (s, 1H), 8.76 (s, 1H), 7.69 (s, 1H), 6.84 (d, *J* = 7.8 Hz, 1H), 6.35 (d, *J* = 7.8 Hz, 1H), 3.41 (s, 3H), 1.35 (s, 9H). ^19^F NMR (376 MHz, CDCl_3_) *δ* −60.32 (s, 9F).

Selected crystal data of *f*‐tpb1: CCDC deposition number: 2104063. C_63_H_69_F_9_IrN_9_O_3_; m = 1363.47; trigonal; space group = P−3; *a* = 17.3685(14) Å, *b* = 17.3685(14) Å, *c* = 14.4365(12) Å; V = 3771.5(7) Å^3^; Z = 2; *ρ*_calcd_ = 1.201 g·cm^−3^; F(000) = 1384; *λ*(Mo−K_*α*_) = 0.71073 Å; T = 243 K; μ = 1.835 mm^−1^; 35 121 reflections collected, 5156 independent reflections (*R*_int_ = 0.0555), data / restraints / parameters = 5156 / 186 / 289, GOF = 1.053, final R_1_[*I* > 2*σ*(*I*)] = 0.0254 and *w*R_2_(all data) = 0.597l largest diff. peak and hole = 0.546 and ‐0.516 e·Å^‒3^.

Synthesis of *mer*‐ and *fac*‐[tris(3‐(3‐tert‐butylphenyl)‐1‐methyl‐6‐(trifluoromethyl)‐3*H*‐1*λ* ^4^‐imidazo[4,5‐b]pyridine‐*C,C^2′^*) iridium(III)] (*m*‐tpb2 and *f*‐tpb2)

Both the complexes *m*‐tpb2 and *f*‐tpb2 were obtained in 53.0% and 26.0% yields as to that described for *m*‐tpb1 and *f*‐tpb1.

Spectral data of *m*‐tpb2: ^1^H NMR (400 MHz, CDCl_3_) *δ* 9.02 (d, *J* = 1.4 Hz, 1H), 8.98 (s, 1H), 8.92 (d, *J* = 1.6 Hz, 1H), 7.63 (s, 2H), 7.55 (s, 2H), 7.49 (d, *J* = 8.3 Hz, 1H), 7.38 (d, *J* = 8.3 Hz, 1H), 6.92 (dd, *J* = 7.7, 1.5 Hz, 1H), 6.85−6.78 (m, 2H), 6.76 (s, 2H), 6.47 (d, *J* = 7.6 Hz, 1H), 3.33 (s, 3H), 3.27 (s, 3H), 3.24 (s, 3H), 1.35 (s, 18H), 1.31 (s, 9H). ^19^F NMR (376 MHz, CDCl_3_) *δ*−66.22 (s, 3F), ‐66.23 (s, 3F), ‐66.26 (s, 3F).

Spectral data of *f*‐tpb2: ^1^H NMR (400 MHz, CDCl_3_) *δ* 8.93 (d, *J* = 1.9 Hz, 1H), 7.44 (d, *J* = 8.3 Hz, 1H), 7.31 (d, *J* = 8.3 Hz, 1H), 6.88 (dd, *J* = 7.8, 1.9 Hz, 1H), 6.40 (d, *J* = 7.8 Hz, 1H), 3.21 (s, 3H), 1.35 (s, 9H). ^19^F NMR (376 MHz, CDCl_3_) *δ−*66.21 (s, 9F).

### Isomerization from *m*‐tpb1 to *f*‐tpb1

To a 100 mL pressure bottle, was added 0.575 g of *m*‐tpb1 (0.480 mmol), 53 mL of ethyl acetate, and 4.9 mL of trifluoroacetic acid (1 m in H_2_O). The mixture was heated to 65 °C for 15 h under N_2_ atmosphere. The progression was indicated by the slow precipitation of the *fac* complex. After cooling to RT, the precipitate was filtered off, washed sequentially with ethyl acetate, water and diethylether and dried under vacuum, giving a grey solid *f*‐tpb1 (0.402 g, 0.336 mmol, conversion yield: 70%), together with unreacted starting material m‐tpb1 (0.121 g, 0.101 mmol). Single crystals of *f*‐tpb1 suitable for X‐ray diffraction study were obtained from a layered solution of acetone and n‐hexane at RT.

### Isomerization from *m*‐tpb2 to *f*‐tpb2

The experimental procedures are akin to those described for *m*‐tpb1, giving a pale‐green solid *f*‐tpb2 (0.431 g, 0.36 mmol, conversion yield: 75%), together with unreacted starting material *m*‐tpb2 (0.103 g, 0.086 mmol).

## Conflict of Interest

The authors declare no conflict of interest.

## Supporting information

Supporting Information

## Data Availability

The data that support the findings of this study are available in the supplementary material of this article.
