# Supplementary material for: Blue Phosphorescence and Hyperluminescence Generated from Imidazo[4,5‐b]pyridin‐2‐ylidene‐Based Iridium(III) Phosphors
Source: Adv Sci (Weinh). 2022 Jul 13;9(25):2201150. doi: 10.1002/advs.202201150 (PMC9443441; doi:10.1002/advs.202201150)
Supplement: Supplementary file 1 — Supporting Information [file ADVS-9-2201150-s001.pdf]

ADVANCED SCIENCE
Open Access

Supporting Information

for *Adv. Sci.*, DOI 10.1002/advs.202201150

Blue Phosphorescence and Hyperluminescence Generated from Imidazo[4,5-b]pyridin-2-ylidene-Based Iridium(III) Phosphors

Xilin Yang, Xiuwen Zhou, Ye-Xin Zhang*, Deli Li, Chensen Li, Caifa You, Tai-Che Chou, Shi-Jian Su*, Pi-Tai Chou* and Yun Chi**

Supporting Information

Blue Phosphorescence and Hyperluminescence Generated from Imidazo[4,5-b]pyridin-2-ylidene Based Iridium(III) Phosphors

Xilin Yang,[a] Xiuwen Zhou,[b,*] Ye-Xin Zhang,[c,*] Deli Li,[a] Chensen Li,[d] Caifa You,[d] Tai-Che Chou,[e] Shi-Jian Su,[a,*] Pi-Tai Chou,[e,*] and Yun Chi,[d,*]

(a) State Key Laboratory of Luminescent Materials and Devices and Institute of Polymer Optoelectronic Materials and Devices, South China University of Technology, Guangzhou 510640, China, E-mail: mssjsu@scut.edu.cn

(b) School of Mathematics and Physics, The University of Queensland, Brisbane, Queensland 4072, Australia, E-mail: x.zhou6@uq.edu.au

(c) Suzhou Joysun Advanced Materials Co., Ltd. Suzhou, Jiangsu 215126, China, E-mail: ethan@wispo.com.cn

(d) Department of Chemistry, Department of Materials Sciences and Engineering, and Center of Super-Diamond and Advanced Films (COSDAF), City University of Hong Kong, Hong Kong SAR, China, E-mail: yunchi@cityu.edu.hk

(e) Department of Chemistry, National Taiwan University, Taipei 10617, Taiwan, E-mail: chop@ntu.edu.tw

Experimental section:

General information and materials. Commercially available reagents were used without further purification. ^{1}H and ^{19}F NMR spectra were measured with a NMR 400MHz instrument (Bruker AVANCE III, BBO probe). Elemental analysis was carried out on an Elemental Micro Carbon-Hydrogen-Nitrogen Analyzer (Elementar VARIO Micro Cube). Mass spectra were recorded on an Applied Biosystems 4800 Plus MALDI TOF/TOF Analyzer using 2,5-dihydroxybenzoic acid as the matrix substance.

Photophysical measurements. UV-Vis absorption spectra were measured with an UV-Visible NIR spectrophotometer system (HITACHI U-3310). The steady-state emission spectra were measured with a spectrofluorometer (Edinburgh FLS920) and the lifetime decay profiles were measured with a time-correlated single photon counting (TCSPC) system coupled with a mode-locked Ti:Sapphire laser (Spectra Physics, Model 3960) followed by a pulse picker (Spectra Physics, Model 3980). The picked pulse then passes through a frequency-doubling crystal (BBO) to generate the excitation source. Lastly, the polarizer between the sample chamber and detector is set at magic angle relative to the excitation source in order to eliminate the polarization effects. All solution samples were degassed using at least three freeze-pump-thaw cycles. Photoluminescence quantum yields in solution at RT are calculated using Coumarin 102 (C102) in methanol (Q.Y. = 0.87) as the standard with corrections on refractive indices of different solvents, while quantum yields in PMMA thin film was measured by an integrated sphere. Lifetimes for thin films were performed by an Edinburgh FLS980 time-correlated single photon counting (TCSPC) system with an EPL-375 diode laser as the excitation source.

Electrochemistry. Cyclic voltammetry was measured with an electrochemical analyzer (CHI660) equipped with a three-electrode system (glassy carbon: working electrode, platinum wire: auxiliary electrode, Ag/AgCl: reference electrode). Nitrogen-purged acetonitrile was used as solvent and NBu_4PF_6 (0.1 M) was used as supporting electrolyte. The potentials were referenced externally to the ferrocenium/ferrocene (Fc^+/Fc) couple.

Computational details. The geometries, electronic structures and electronic excitations of the studied Ir(II) complexes were firstly investigated by methods based on DFT[1] and

TD-DFT[2] using the B3LYP functional[3] with Gaussian 16 set of programs.[4] The polarizable continuum model (PCM)[5] was used to include solvent effects of toluene. The 6-31G(d,p)[6] basis set was used for light elements such as hydrogen, carbon, nitrogen, oxygen and fluorine, while the LANL2DZ[7] basis set with the Los Alamos National Laboratory (LANL) effective core potentials (ECPs) was used for iridium. The corresponding ground state (S_0) geometries were optimized based on the X-ray structural data of ***f*-tpb1** and ***f*-tpb2**. The low-energy excited states were then calculated by using the TD–DFT method based on the optimized ground state structures. Avogadro software[8] was used to visualize the orbitals presented in this work. Orbital composition analysis was performed using the Hirshfeld method[9] to calculate the contribution of metal atom to each molecular orbital with the Multiwfn[10] software.

Relativistic TD-DFT calculations were further performed to obtain more detailed information on spin-mixed electron excitations (e.g., the oscillator strength and the radiative rate for $S_0 \rightarrow T_1$ excitations) with ADF (2019 version)[11] using the B3LYP functional and Slater type TZP[12] basis sets. The COnductor-like Screening MOdel (COSMO)[13] is used to model solvent effects.

In these calculations, spin-orbit coupling (SOC) was added perturbatively[14] to one-component TDDFT[2a] utilizing the one-component zeroth order regular approximation (ZORA).[15] A total of 24 spin-mixed excitations were calculated. The radiative rate (k_r) of an excited state was then calculated according to[16]

$$k_r = 2\epsilon^2 f / c^3 \qquad (1)$$

where ϵ is the excitation energy and f is the corresponding oscillate strength for the electronic excitation from the ground state to excited state, and the equation is in atomic units with *c* representing the speed of light in vacuum. The three lowest energy states found in the SOC TDDFT calculations for the complexes considered here are derived from the three substrates in a triplet state with a small singlet admixture due to SOC.[17] There will be small energy differences between the three states because of their different admixtures with the singlet state, the so called zero-field splitting (ZFS). As the ZFS is small compared to $k_B T$ at room temperature, the radiative rate of the triplet state is then calculated as an average over the three substrates within the assumption of fast thermalization.

Device fabrication and measurement. Devices were fabricated on patterned ITO glass

substrates with a sheet resistance of 15 Ω per square. Before device fabrication, ITO substrates were cleaned with tetrahydrofuran, deionized water and isopropanol in sequence, dried in an oven for at least 2 h, treated with UV-ozone for 10 min, and finally loaded into a deposition chamber with a basic pressure less than 5×10^{-4} Pa. Deposition rates and thicknesses of all materials were monitored with oscillating quartz crystals. The deposited rates for organic materials, LiF, and Al were controlled at 1 – 2, 0.1, and 7 Å s^{-1}, respectively. The current density-voltage (*J*-*V*-*L*) characteristics of the devices were measured using a constant current source (Keithley 2400 Sourcemeter) combined with a photometer (Spectrophotometer CS 200). The electroluminescence spectra were collected by the optical fiber spectrometer (USB 2000+). All measurements were performed at RT without encapsulation.

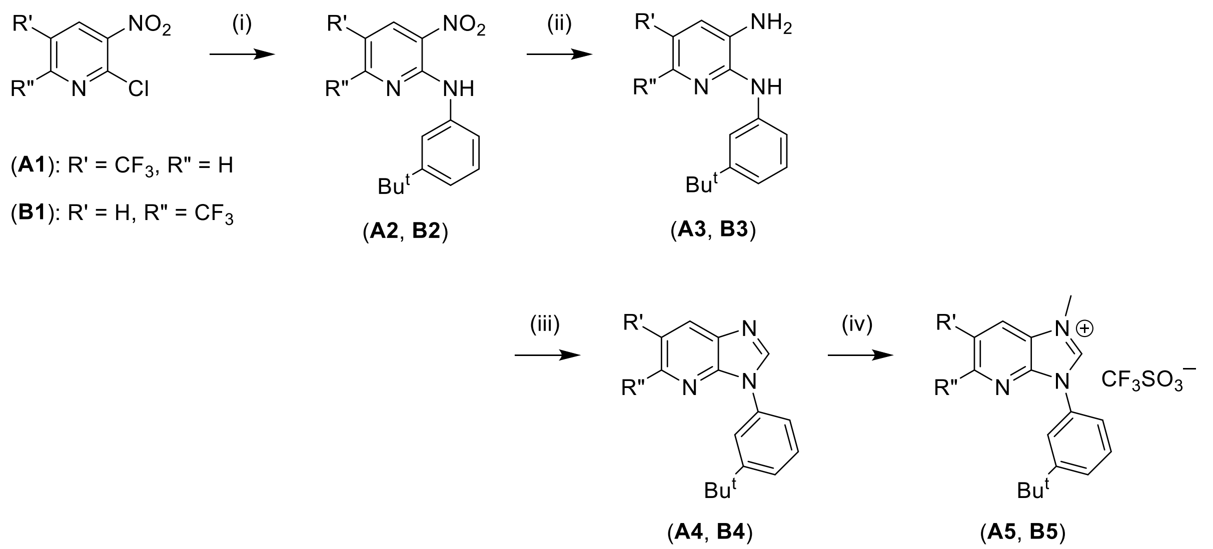

Scheme S1. Synthetic route to studied carbene chelates: (i) 3-*tert*-butylaniline, ethylene glycol, 150 °C; (ii) NH_4Cl, Fe, MeOH, H_2O, THF, 70 °C; (iii) formic acid, 70 °C; (iv) $CF_3SO_3CH_3$, RT.

Synthesis of 2-chloro-3-nitro-6-(trifluoromethyl)pyridine (**B1**):

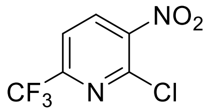

To a mixture of concentrated sulfuric acid (75 mL) and hydrogen peroxide (40%, 37 mL) was added dropwise to a solution of 2-chloro-6-(trifluoromethyl)-3-pyridinamine (7.00 g, 35.6 mmol) in sulfuric acid (93 mL) at 0 °C. The reaction mixture was stirred at RT for 20 hours. After then, the mixture was poured into an ice-water mixture and neutralized to pH 8-9 by addition of conc. NH_4OH (aq) and extracted with ethyl acetate. The combined organic layers were dried over anhydrous Na_2SO_4 and concentrated to give a yellow solid (3.72 g). ^1H NMR (400 MHz, $CDCl_3$) δ 8.4 (d, 1H), 7.8 (d, 1H).

Synthesis of *N*-(3-*tert*-butylphenyl)-3-nitro-5-(trifluoromethyl)pyridin-2-amine (**A2**):

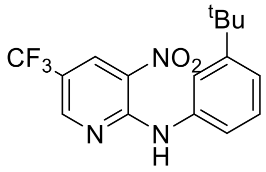

A mixture of 2-chloro-3-nitro-5-(trifluoromethyl)pyridine (2.26 g, 10 mmol), 3-*tert*-butylaniline (1.49 g, 10 mmol) in ethylene glycol (40 mL) was placed into a seal-tube

and heated at 150 °C for 12 h. After cooled to RT, water (50 mL) was added and organic material was extracted into ethyl acetate (50 mL), washed with water and brine in sequence, dried over anhydrous Na_2SO_4 and, concentrated by rotatory evaporation. The crude product was further purified via silica gel column chromatography using hexane/ethyl acetate (5/1, v/v) as the eluent to afford a red solid; yield: 2.95 g, 87%. ^{1}H NMR (400 MHz, $CDCl_3$) δ 10.27 (s, 1H), 8.77 (s, 1H), 8.70 (s, 1H), 7.52 (d, *J* = 7.1 Hz, 1H), 7.52 (s, 1H), 7.39 (t, *J* = 8.0 Hz, 1H), 7.32 (d, *J* = 7.9 Hz, 1H), 1.37 (s, 9H).

Synthesis of *N*-(3-*tert*-butylphenyl)-3-nitro-6-(trifluoromethyl)pyridin-2-amine (**B2**):

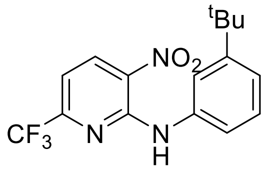

This compound was prepared similarly as **B1**, giving a red solid with yield: 2.98 g (88%). ^{1}H NMR (400 MHz, $CDCl_3$) δ 10.21 (s, 1H), 8.72 (d, *J* = 8.4 Hz, 1H), 7.93 (s, 1H), 7.42 (d, *J* = 8.8 Hz, 1H), 7.35 (t, *J* = 7.8 Hz, 1H), 7.25 (d, *J* = 7.7 Hz, 1H), 7.17 (d, *J* = 8.5 Hz, 1H), 1.37 (s, 9H).

Synthesis of *N*^{2}-(3-*tert*-butylphenyl)-5-(trifluoromethyl)pyridine-2,3-diamine (**A3**):

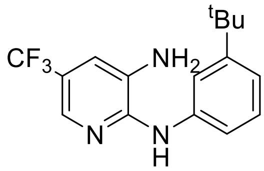

The mixture of **A2** (3.39 g, 10 mmol), NH_4Cl (1.34 g, 25 mmol) and Fe (1.4 g, 25 mmol) in a mixture of H_2O (40 mL), MeOH (20 mL) and THF (20 mL) was stirred for 8 h at 70 °C and, then, cooled to RT. The mixture was filtered through Celite® and solution was evaporated to dryness. The residue was next dissolved in ethyl acetate and washed with distilled water (100 mL). The organic layer was separated and concentrated to dryness. The crude product was purified by column chromatography using petroleum ether/ethyl acetate (3/1, v/v) as eluent to give a gray solid. Yield: 2.60 g (84%). ^{1}H NMR (400 MHz, $CDCl_3$) δ 8.11 (s, 1H), 7.42 – 7.35 (m, 3H), 7.31 (d, *J* = 7.7 Hz, 1H), 7.17 (d, *J* = 1.6 Hz, 1H), 7.12 (d, *J* = 7.7 Hz, 1H), 6.56 (s, 1H), 3.48 (s, 2H), 1.35 (s, 9H).

Synthesis of *N*^{2}-(3-*tert*-butylphenyl)-6-(trifluoromethyl)pyridine-2,3-diamine (**B3**):

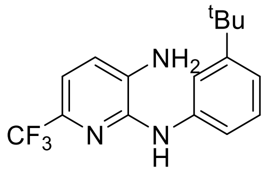

This compound was prepared similarly as **A3**, giving a gray solid with yield: 2.66 g (86%). ^{1}H NMR (400 MHz, $CDCl_3$) δ 7.65 (s, 1H), 7.25 (d, *J* = 7.8 Hz, 1H), 7.17 – 7.10 (m, 2H), 7.10 – 7.01 (m, 2H), 6.40 (s, 1H), 3.44 (s, 2H), 1.35 (s, 9H).

Synthesis of 3-(3-*tert*-butylphenyl)-6-(trifluoromethyl)-3*H*-imidazo[4,5-*b*]pyridine (**A4**):

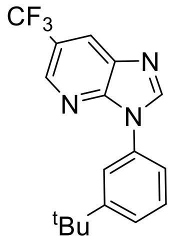

A solution of **A3** (3.09 g, 10 mmol) in 50 mL formic acid was heated to 100 °C for 12 h. After cooled to RT, the remaining formic acid was evaporated and residue was extracted into ethyl acetate (50 mL), washed with water, dried over anhydrous Na_2SO_4 and, then, concentrated by rotatory evaporation. The crude product was further purified via silica gel column chromatography using hexane/ethyl acetate (5/1, v/v) as the eluent to afford a gray solid; yield: 3.0 g, 94%. ^{1}H NMR (400 MHz, $CDCl_3$) δ 8.76 (s, 1H), 8.47 (s, 1H), 8.42 (s, 1H), 7.70 (s, 1H), 7.55 (s, 3H), 1.41 (s, 9H).

Synthesis of 3-(3-*tert*-butylphenyl)-5-(trifluoromethyl)-3*H*-imidazo[4,5-*b*]pyridine (**B4**):

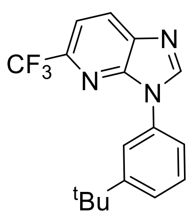

This compound was prepared similarly as **A4**, giving a gray solid with yield: 2.9 g (91%). ^{1}H NMR (400 MHz, $CDCl_3$) δ 8.56 (s, 1H), 8.30 (d, *J* = 8.3 Hz, 1H), 7.92 (s, 1H), 7.74 (d, *J* = 8.3 Hz, 1H), 7.60 – 7.53 (m, 2H), 7.52 (t, *J* = 1.9 Hz, 1H), 1.42 (s, 9H).

Synthesis of

3-(3-(tert-butyl)phenyl)-1-methyl-6-(trifluoromethyl)imidazo[4,5-b]pyridin-1-ium trifluoromethanesulfonate (**A5**):

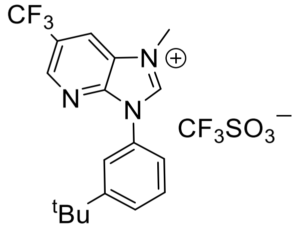

A toluene (40 mL) solution of **A4** (2.55 g, 8 mmol) was slowly added methyl trifluoromethanesulfonate (1.72 g, 1.19 mL, 10.5 mmol) and the reaction mixture was stirred at RT for 4 h. The resulting precipitate was filtered off, washed with ethyl ether, and dried under vacuum to provide a white solid. Yield: 2.25 g (88%). ^{1}H NMR (400 MHz, acetone-d_6) δ 10.49 (s, 1H), 9.26 (s, 1H), 9.24 (s, 1H), 8.06 (t, *J* = 2.0 Hz, 1H), 7.86 - 7.79 (m, 2H), 7.72 (t, *J* = 7.9 Hz, 1H), 4.56 (s, 3H), 1.42 (s, 9H).

Synthesis of 3-(3-(tert-butyl)phenyl)-1-methyl-5-(trifluoromethyl)imidazo[4,5-b]pyridin-1-ium trifluoromethanesulfonate (**B5**):

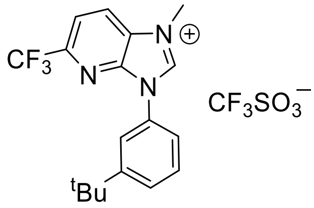

This compound was prepared similarly as **A5**, giving a yellow solid with yield: 2.3 g (90%). ^{1}H NMR (400 MHz, acetone-d_6) δ 10.54 (s, 1H), 9.07 (d, *J* = 8.6 Hz, 1H), 8.42 (d, *J* = 8.6 Hz, 1H), 8.11 (t, *J* = 1.9 Hz, 1H), 7.84 (dd, *J* = 7.8 Hz, 1.0 Hz, 1H), 7.81 (dd, *J* = 8.5 Hz, 1.1 Hz, 1H), 7.72 (t, *J* = 7.9 Hz, 1H), 4.56 (s, 3H), 1.42 (s, 9H).

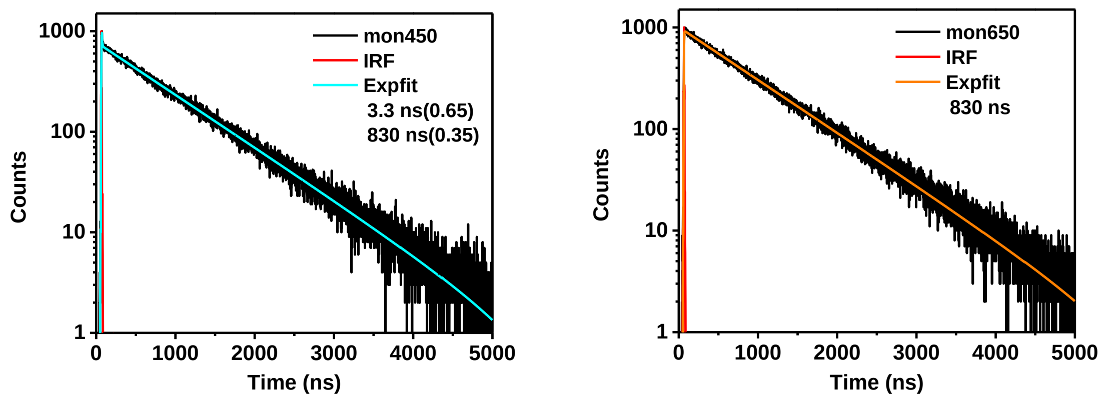

Figure S1. Transient decay spectra of ***m*-tpb1** at 450 and 650 nm measured in degassed toluene at RT (10^{-5} M).

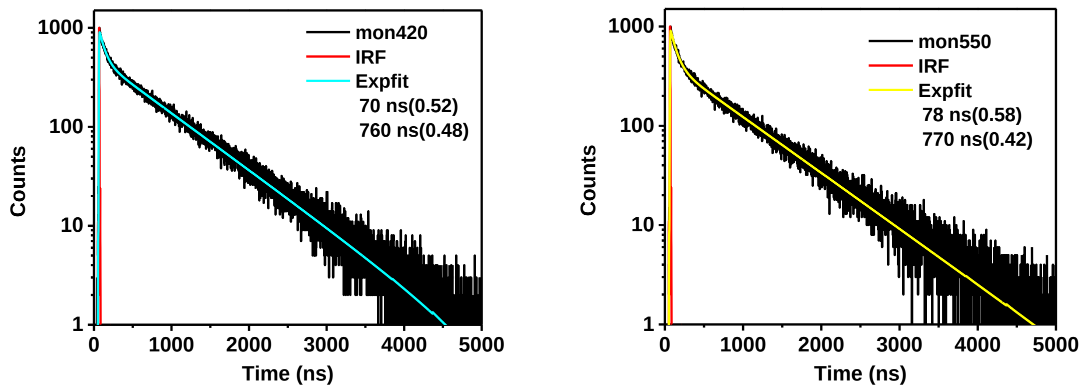

Figure S2. Transient decay spectra of ***f*-tpb1** at 420 and 550 nm measured in degassed toluene at RT (10^{-5} M)

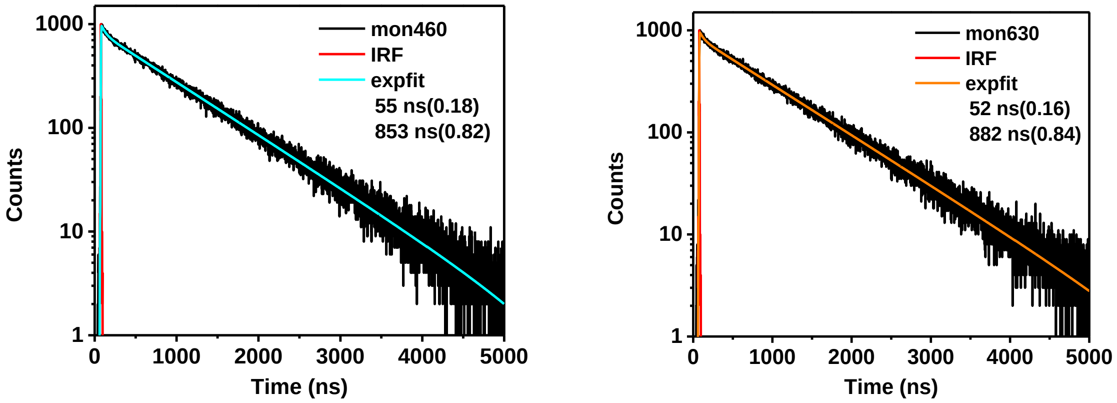

Figure S3. Transient decay spectra of ***m*-tpb2** at 460 and 630 nm measured in degassed toluene at RT (10^{-5} M).

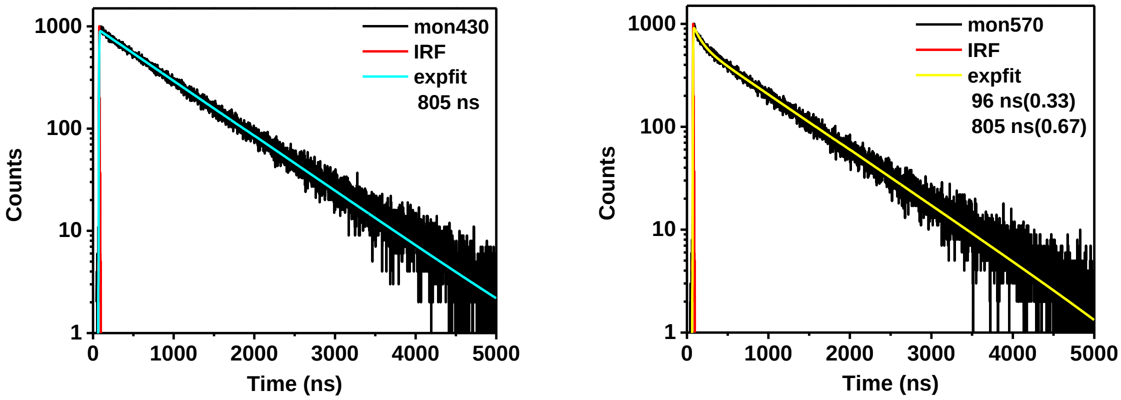

Figure S4. Transient decay spectra of ***f*-tpb2** at 430 and 570 nm measured in degassed toluene at RT (10^{-5} M).

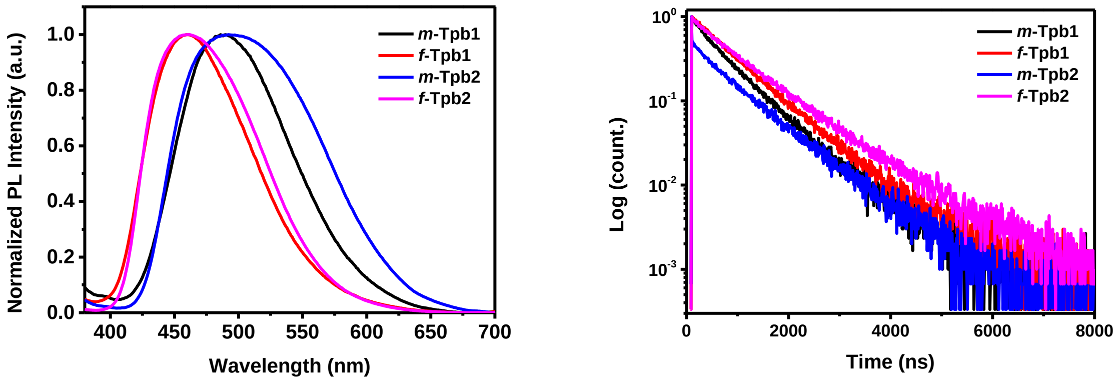

Figure S5. Photoluminescence spectra and lifetimes of the studied tris-bidentate Ir(III) carbene complexes measured in PMMA thin films at 2 wt% at RT.

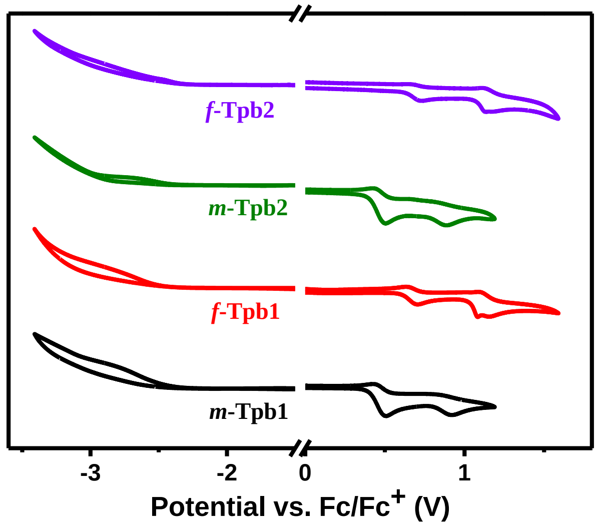

Figure S6. Cyclic voltammograms of all studied tris-bidentate Ir(III) complexes in acetonitrile solution.

	***f*-tpb1**	***f*-tpb2**
LUMO+1	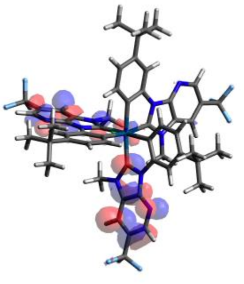 -1.59 eV, Ir: 4.1%	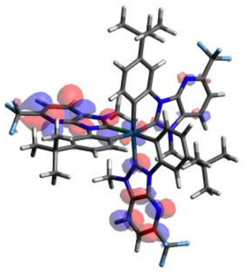 -1.62 eV, Ir: 4.0%

Figure S7. LUMO+1 of Ir(III) complexes ***f*-tpb1** and ***f*-tpb2** at their geometries optimized for the ground state. The orbital energy level and the contribution from Ir atom are also provided. Note the HOMO to LUMO+1 orbital transition contributes dominantly to the S_1 excited states for ***f*-tpb1** and ***f*-tpb2** (*cf*. **Table 3**).

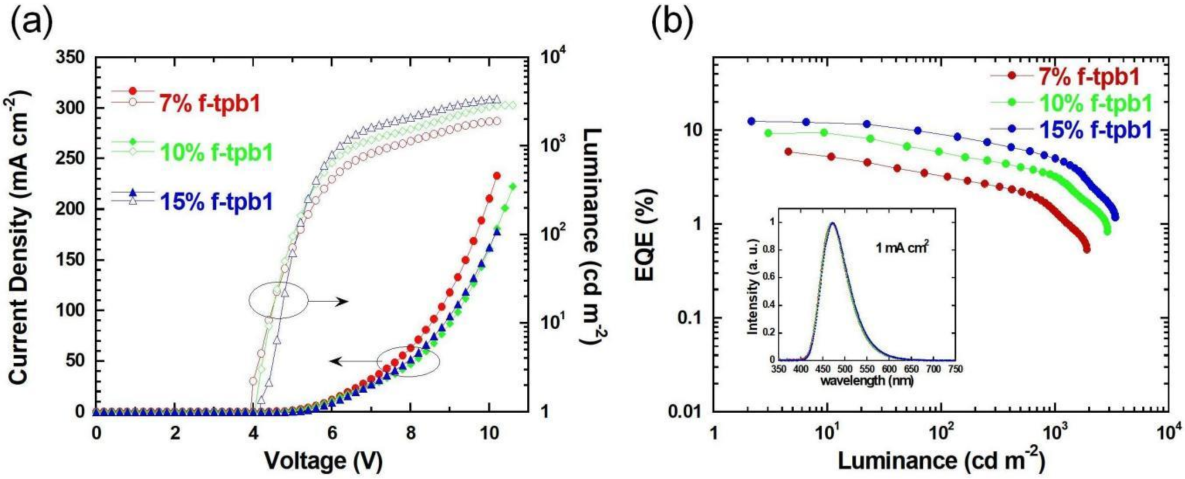

Figure S8. Device characteristics based on Ir(III) complex ***f*-tpb1** with different doping concentrations. (a), Current density−voltage−luminance characteristics; (b), EQE to current density curve with EL spectra inserted.

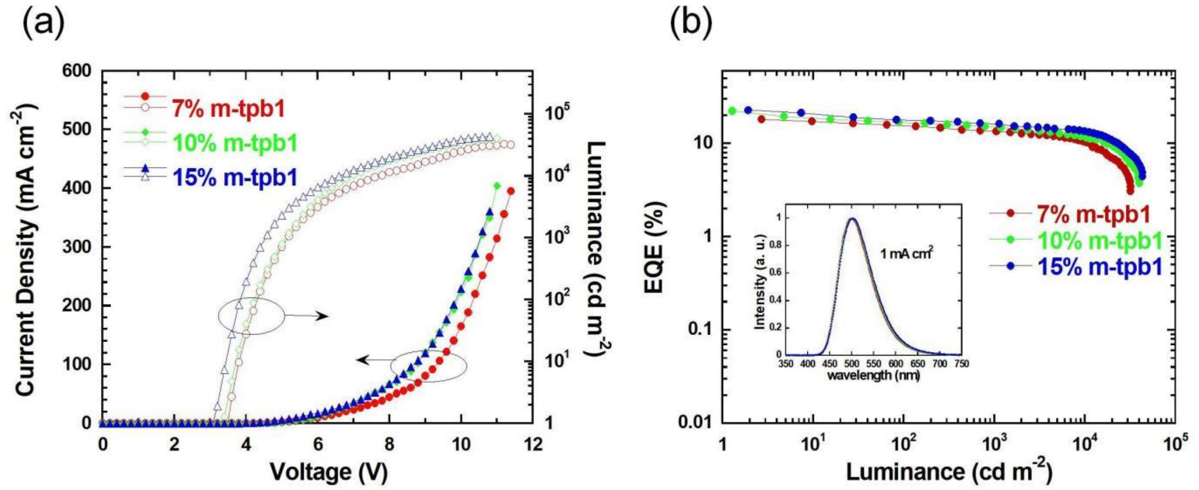

Figure S9. Device characteristics based on Ir(III) complex ***m*-tpb1** with different doping concentrations. (a), Current density−voltage−luminance characteristics; (b), EQE to current density curve with EL spectra inserted.

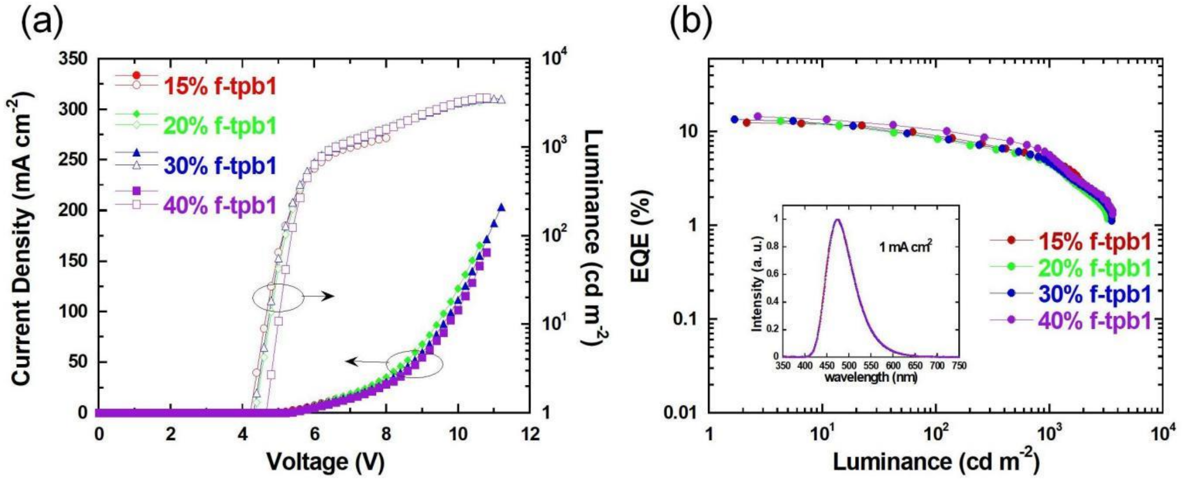

Figure S10. Device characteristics based on Ir(III) complex ***f*-tpb1** with different doping concentrations. (a), Current density−voltage−luminance characteristics; (b), EQE to current density curve with EL spectra inserted.

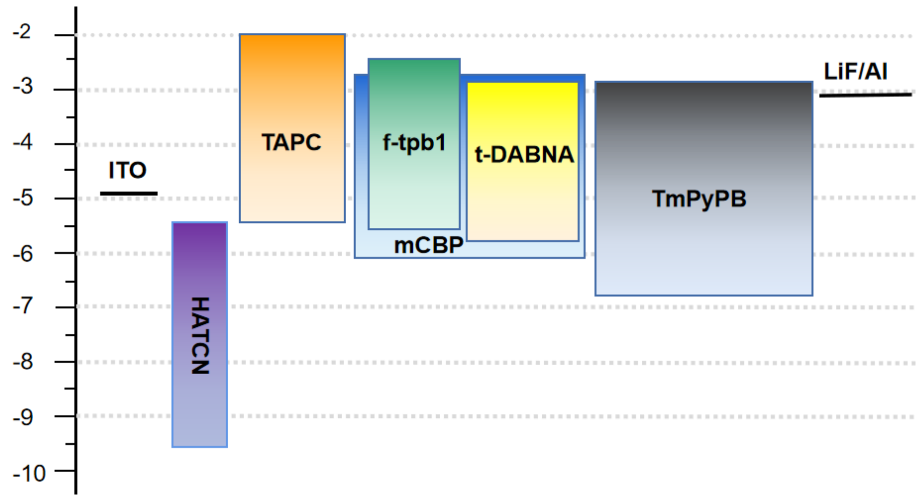

Figure S11. Hyperphosphorescence OLED device structure using ***f*-tpb1** as sensitizer and *t*-DABNA as terminal emitter.

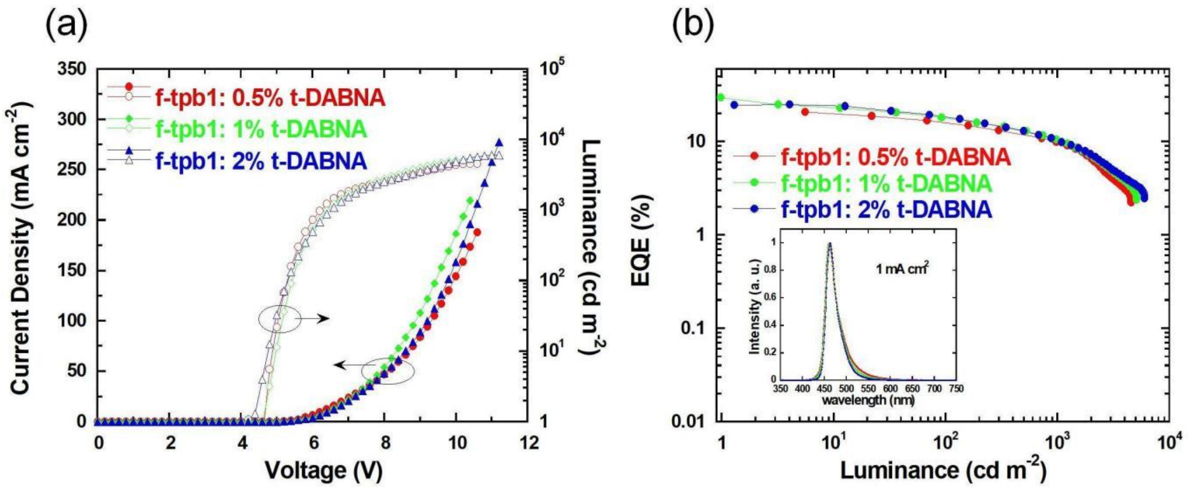

Figure S12. The performance of phosphor sensitized hyperphosphorescence devices using mCBP: 30% ***f*-tpb1**: y% *t*-DABNA as emitting layer (EML), y is varied from 0.5% to 2%. (a), Current density−voltage−luminance characteristics; (b), EQE to current density curve with EL spectra inserted.

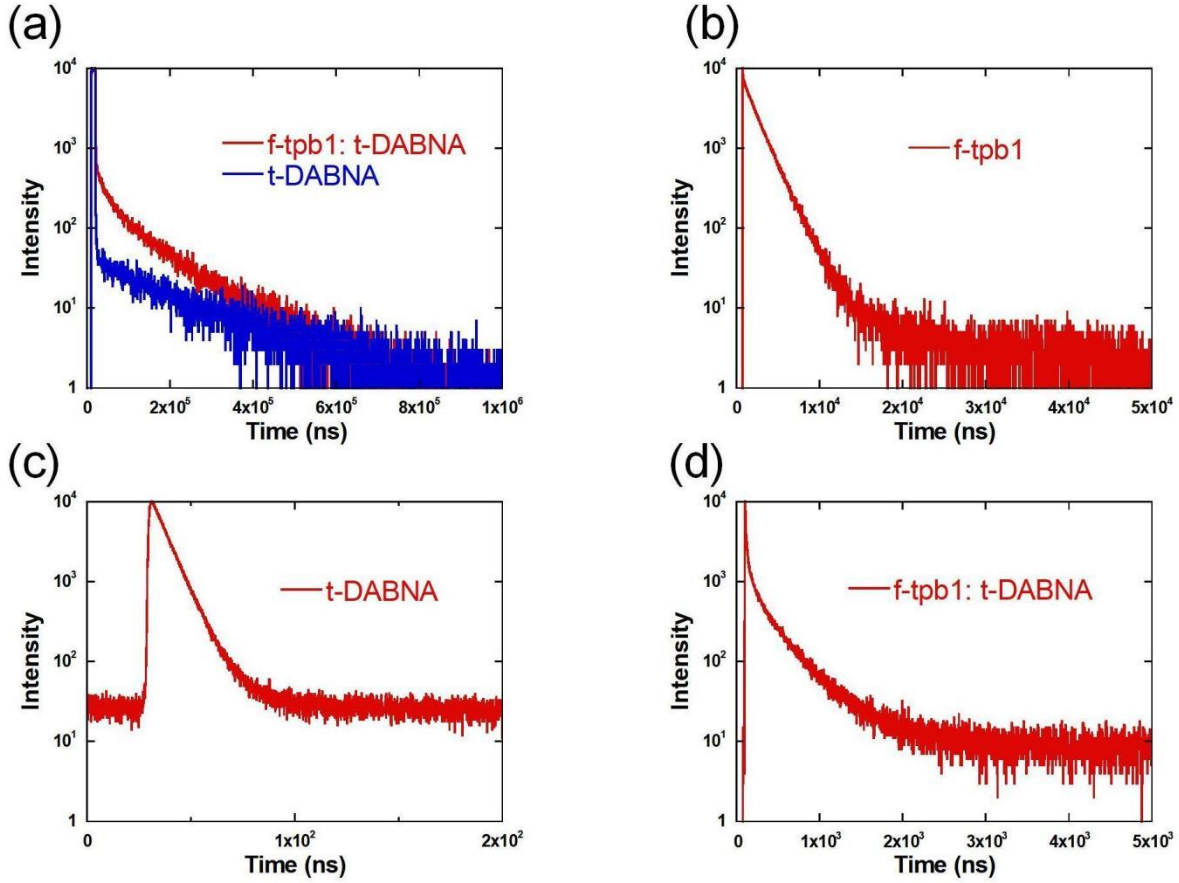

Figure S13. (a), (b) Long-time scale and (c), (d) short-time scale components of the transient PL decays of the films of mCBP: 30% ***f*-tpb1**, mCBP: 1% *t*-DABNA, and mCBP: 30% ***f*-tpb1**: 1% *t*-DABNA (50 nm thickness) in air.

Table S1. Summarized photophysical data of these complexes in PMMA thin films.

complex	PL λ_{max} [nm] [(a)]	FWHM (cm^{-1} / nm) [(b)]	Φ_P (%)	τ_{obs} [μs] [(a)]
***m*-Tpb1**	490	3641 / 103	58	0.757
***f*-Tpb1**	460	3497 / 95	63	0.797
***m*-Tpb2**	492	3779 / 131	60	0.879
***f*-Tpb2**	461	3780 / 101	58	0.963

(a) Measured in PMMA thin films at 2 wt.% at RT; (b) FWHM: full width at half maxima of PL emission peak max. in both cm^{-1} and nm.

Table S2. The relativistic calculation results for the excitation energy (ϵ) and corresponding oscillator strength (f), calculated radiative decay rate (k_r), and the assignment of charge characters of the lowest triplet (T_1) excited states for the six studied Ir(III) complexes at their geometries optimized for the ground state.

	ϵ [a]	f (10^{-5}) [a]	k_r (10^4 s^{-1}) [b]
***m*-tpb1**	449 nm / 2.76 eV	6.4	2.1
***f*-tpb1**	430 nm / 2.89 eV	7.4	2.7
***m*-tpb2**	459 nm / 2.70 eV	4.9	1.5
***f*-tpb2**	439 nm / 2.83 eV	6.3	2.2

[a] The calculated ϵ, f, and k_r of T_1 state are averaged from the three substates of T_1 state, where spin-mixed excitations were calculated by relativistic calculations with spin−orbit coupling (SOC) added perturbatively to one-component TD-DFT, and with COSMO for modeling the toluene solvent (see computational details for more information).

[b] Note that the calculated k_r (at 0 K) is systematically smaller than the experimental k_r (at RT), same as that found in other families of Ir(III) complexes. The former is intrinsic and independent from the non-radiative decay pathways, whereas the latter is highly dependent on the non-radiative decay pathways. The uncertainty of applied computational model and methods is also one of possible sources of deviation between calculated and experimental k_r. In general, the trend of calculated and experimental k_r is consistent.

Table S3. Summarized EL properties of phosphorescent OLED devices with emitter ***f*-tpb1** as emitter at different doping concentrations.

EML	V_{on} (a) [V]	L_{max} [cd m^{-2}]	max. EQE / CE / PE [% / cd A^{-1} / lm W^{-1}]	λ_{max} [nm]	FWHM(b) [nm]	CIE [x, y]
mCBP: 7% ***f*-tpb1**	4	1913	5.9 / 9.0 / 6.8	470	71	0.15, 0.20
mCBP: 10% ***f*-tpb1**	4.1	2897	9.5 / 14.8 / 10.6	472	71	0.15, 0.21
mCBP: 15% ***f*-tpb1**	4.2	3397	12.5 / 20.3 / 14.5	472	71	0.15, 0.22
mCBP: 20% ***f*-tpb1**	4.4	3289	13.3 / 22.4 / 16	472	73	0.15, 0.23
mCBP: 30% ***f*-tpb1**	4.4	3558	13.5 / 22.8 / 16.3	472	73	0.15, 0.23
mCBP: 40% ***f*-tpb1**	4.7	3629	14.5 / 25.1 / 16.4	476	74	0.16, 0.24

a) Data recorded at 1 cd m^{-2}. (b) Full width at half maximum of EL spectrum.

Table S4. Summarized EL properties of phosphorescent OLED devices with emitter ***m*-tpb1** at different doping concentrations.

EML	V_{on} (a) [V]	L_{max} [cd m^{-2}]	max. EQE / CE / PE [% / cd A^{-1} / lm W^{-1}]	λ_{max} [nm]	FWHM(b) [nm]	CIE [x, y]
mCBP: 7% ***m*-tpb1**	3.5	33390	18.2 / 47.7 / 41.6	498	86	0.21, 0.44
mCBP: 10% ***m*-tpb1**	3.4	40061	22.3 / 59.3 / 54.8	501	87	0.21, 0.45
mCBP: 15% ***m*-tpb1**	3.2	43261	22.8 / 61.7 / 60.6	503	88	0.22, 0.45

(a) Data recorded at 1 cd m^{-2}. (b) Full width at half maximum of EL spectrum.

Table S5. Summarized EL properties of the hyperphosphoresent devices with different doping concentrations of terminal emitter *t*-DABNA and the reference device with emitter *t*-DABNA.

EML	V_{on} [(a)] [V]	L_{max} [cd m^{-2}]	max. EQE / CE / PE [% / cd A^{-1} / lm W^{-1}]	λ_{max} [nm]	FWHM[(b)] [nm]	CIE [x, y]
mCBP: 30% ***f*-tpb1**: 0.5% *t*-DABNA	4.7	4547	20.8 / 22.6 / 14.8	461	31	0.14, 0.14
mCBP: 30% ***f*-tpb1**: 1% *t*-DABNA	4.6	5084	29.6 / 28.7 / 19.6	462	30	0.13, 0.11
mCBP: 30% ***f*-tpb1**: 2% *t*-DABNA	4.4	5983	25.0 / 21.9 / 15.0	463	27	0.13, 0.10
mCBP: 1% *t*-DABNA	4.1	3886	14.2 / 12.8 / 9.6	463	26	0.14, 0.10

[(a)] Data recorded at 1 cd m^{-2}; [(b)] Full width at half maximum of EL spectrum.

Table S6. Key performance data for some reported deep blue devices (CIE_y < 0.16) with phosphor sensitizer and TADF terminal emitter.

Sensitizer	Emitter	Host	Max. EQE [%]	λ_{max} [nm]	FWHM[a] [nm]	CIE [x, y]	Refs
m-tz2	*t*-DABNA	DPEPO	19.7	468	31	0.12, 0.13	[18]
PtON7-dtb	*v*-DABNA	oCBP: mCBP-2CN	32.2	473	20	0.11, 0.14	[19]
CN-Ir	*v*-DABNA	mCBP: SiCz2Trz	27.3	470	20	0.13 0.16	[20]
Ir(cb)3	*t*-DABNA	TSPO1	24.8	464	27	0.13, 0.11	[21]
***f*-tpb1**	*t*-DABNA	mCBP	29.6	462	30	0.13, 0.11	This work

[(a)] Full width at half maximum of EL spectrum.

Table S7. The exciton lifetimes of the films (50 nm) of mCBP: 30% ***f*-tpb1**, mCBP: 1% *t*-DABNA, and mCBP: 30% ***f*-tpb1**: 1% *t*-DABNA.

film	Prompt lifetime	Delayed lifetime [μs]	
	τ_1 [ns]	τ_2 [μs]	τ_3 [μs]
mCBP: 30% ***f*-tpb1**	–	1.8 (100%)	–
mCBP: 1% *t*-DABNA	7.1 (74%)	171.4 (26%)	–
mCBP: 30% ***f*-tpb1**: 1% *t*-DABNA	12.1 (6%)	0.5 (21%)	94.9 (73%)

[a] Decays of the exciton lifetimes were obtained by the multi-exponential fitting of the decay curves of the phosphor sensitized films, and number in parenthesis was the relative percentage.

References

[1] a) W. Kohn, L. J. Sham, *Phys. Rev.* **1965**, *140*, A1133-A1138; b) P. Hohenberg, W. Kohn, *Phys. Rev.* **1964**, *136*, B864-B871.

[2] a) M. E. Casida, *J. Mol. Struct. THEOCHEM* **2009**, *914*, 3-18; b) E. Runge, E. K. U. Gross, *Phys. Rev. Lett.* **1983**, *52*, 997-1000.

[3] a) P. J. Stephens, F. J. Devlin, C. F. Chabalowski, M. J. Frisch, *J. Phys. Chem.* **1994**, *98*, 11623-11627; b) A. D. Becke, *J. Chem. Phys.* **1993**, *98*, 5648-5652; c) C. Lee, W. Yang, R. G. Parr, *Phys. Rev. B* **1988**, *37*, 785-789.

[4] M. J. Frisch, G. W. Trucks, H. B. Schlegel, G. E. Scuseria, M. A. Robb, J. R. Cheeseman, G. Scalmani, V. Barone, B. Mennucci, G. A. Petersson, H. Nakatsuji, M. Caricato, X. Li, H. P. Hratchian, A. F. Izmaylov, J. Bloino, G. Zheng, J. L. Sonnenberg, M. Hada, M. Ehara, K. Toyota, R. Fukuda, J. Hasegawa, M. Ishida, T. Nakajima, Y. Honda, O. Kitao, H. Nakai, T. Vreven, J. A. Montgomery, J. E. Peralta, F. Ogliaro, M. Bearpark, J. J. Heyd, E. Brothers, K. N. Kudin, V. N. Staroverov, R. Kobayashi, J. Normand, K. Raghavachari, A. Rendell, J. C. Burant, S. S. Iyengar, J. Tomasi, M. Cossi, N. Rega, J. M. Millam, M. Klene, J. E. Knox, J. B. Cross, V. Bakken, C. Adamo, J. Jaramillo, R. Gomperts, R. E. Stratmann, O. Yazyev, A. J. Austin, R. Cammi, C. Pomelli, J. W. Ochterski, R. L. Martin, K. Morokuma, V. G. Zakrzewski, G. A. Voth, P. Salvador, J. J. Dannenberg, S. Dapprich, A. D. Daniels, Ö. Farkas, J. B. Foresman, J. V. Ortiz, J. Cioslowski, D. J. Fox, *Gaussian 16, Revision A03; Gaussian Inc.* **2016**, Wallingford, CT.

[5] S. Miertuš, E. Scrocco, J. Tomasi, *Chem. Phys.* **1981**, *55*, 117-129.

[6] P. C. Hariharan, J. A. Pople, *Theor. Chim. Acta* **1973**, *28*, 213-222.

[7] P. J. Hay, W. R. Wadt, *J. Chem. Phys.* **1985**, *82*, 299-310.

[8] M. D. Hanwell, D. E. Curtis, D. C. Lonie, T. Vandermeersch, E. Zurek, G. R. Hutchison, *J. Cheminfor.* **2012**, *4*, 17.

[9] F. L. Hirshfeld, *Theo. Chim. Acta* **1977**, *44*, 129-138.

[10] T. Lu, F. Chen, *J. Comput. Chem.* **2012**, *33*, 580-592.

[11] G. te Velde, F. M. Bickelhaupt, E. J. Baerends, C. Fonseca Guerra, S. J. A. van Gisbergen, J. G. Snijders, T. Ziegler, *J. Comput. Chem.* **2001**, *22*, 931-967.

[12] a) E. Van Lenthe, E. J. Baerends, *J. Comput. Chem.* **2003**, *24*, 1142-1156; b) D. P. Chong, *Mol. Phys.* **2005**, *103*, 749-761.

[13] A. Klamt, G. Schuurmann, *J. Chem. Soc., Perkin Trans. 2* **1993**, 799-805.

[14] F. Wang, T. Ziegler, *J. Chem. Phys.* **2005**, *123*, 154102.

[15] a) C. Chang, M. Pelissier, P. Durand, *Phys. Scr.* **1986**, *34*, 394-404; b) E. van Lenthe, E. J. Baerends, J. G. Snijders, *J. Chem. Phys.* **1994**, *101*, 9783-9792.

[16] a) S. J. Strickler, R. A. Berg, *J. Chem. Phys.* **1962**, *37*, 814-822; b) K. Nozaki, *J. Chin. Chem. Soc.* **2006**, *53*, 101-112; c) X. Zhou, B. J. Powell, *Inorg. Chem.* **2018**, *57*, 8881-8889.

[17] a) B. J. Powell, *Coord. Chem. Rev.* **2015**, *295*, 46-79; b) B. J. Powell, *Sci. Rep.* **2015**, *5*, 10815.

[18] C. You, X.-Q. Wang, X. Zhou, Y. Yuan, L.-S. Liao, Y.-C. Liao, P.-T. Chou, Y. Chi, *ACS Appl. Mater. Interfaces* **2021**, *13*, 59023–59034.

[19] S. Nam, J. W. Kim, H. J. Bae, Y. M. Maruyama, D. Jeong, J. Kim, J. S. Kim, W.-J. Son, H. Jeong, J. Lee, S.-G. Ihn, H. Choi, *Adv. Sci.* **2021**, *8*, 2100586.

[20] W. J. Chung, K. H. Lee, M. Jung, K. M. Lee, H. C. Park, M.-S. Eum, J. Y. Lee, *Adv. Opt. Mater.* **2021**, *9*, 2100203.

[21] K. H. Lee, J. Y. Lee, *J. Mater. Chem. C* **2019**, *7*, 8562-8568.
